# Chemicals Possessing a Neurotrophin-Like Activity on Dopaminergic Neurons in Primary Culture

**DOI:** 10.1371/journal.pone.0006215

**Published:** 2009-07-10

**Authors:** Fanny Schmidt, Pierre Champy, Blandine Séon-Méniel, Xavier Franck, Rita Raisman-Vozari, Bruno Figadère

**Affiliations:** 1 Centre National de la Recherche Scientifique, Laboratoire de Pharmacognosie, Université Paris-Sud 11, Faculté de Pharmacie, Châtenay-Malabry, France; 2 Institut National de la Santé et de la Recherche Médicale, Unité Mixte de Recherche S679 Thérapeutique expérimentale de la neurodégénérescence, Centre de Recherche de L'institut du Cerveau et de la Moëlle, Université Pierre et Marie Curie, Paris, France; 3 Centre National de la Recherche Scientifique, Université de Rouen, Institut National des Sciences Appliquées de Rouen, Unité Mixte de Recherche 6014, COBRA-IRCOF, Mont-Saint-Aignan, France; University of Nebraska, United States of America

## Abstract

**Background:**

Neurotrophic factors have been shown to possess strong neuroprotective and neurorestaurative properties in Parkinson's disease patients. However the issues to control their delivery into the interest areas of the brain and their surgical administration linked to their unability to cross the blood brain barrier are many drawbacks responsible of undesirable side effects limiting their clinical use. A strategy implying the use of neurotrophic small molecules could provide an interesting alternative avoiding neurotrophin administration and side effects. In an attempt to develop drugs mimicking neurotrophic factors, we have designed and synthesized low molecular weight molecules that exhibit neuroprotective and neuritogenic potential for dopaminergic neurons.

**Principal Findings:**

A cell-based screening of an in-house quinoline-derived compound collection led to the characterization of compounds exhibiting both activities in the nanomolar range on mesencephalic dopaminergic neurons in spontaneous or 1-methyl-4-phenylpyridinium (MPP^+^)-induced neurodegeneration. This study provides evidence that rescued neurons possess a functional dopamine transporter and underlines the involvement of the extracellular signal-regulated kinase 1/2 signaling pathway in these processes.

**Conclusion:**

Cell-based screening led to the discovery of a potent neurotrophic compound possessing expected physico-chemical properties for blood brain barrier penetration as a serious candidate for therapeutic use in Parkinson disease.

## Introduction

Loss of dopaminergic (DA) neurons within the substantia nigra pars compacta (SNpc) is a consistent feature of Parkinson's disease (PD). This is mainly clinically characterized by motor impairments [1]. Except for some cases linked to specific gene defects (<10%), PD is a sporadic combination of unknown factors [2]. Mitochondrial dysfunction, oxidative stress, and proteasome failure are among the several hypotheses put forward to explain the molecular basis of neuronal damage [3], [4]. The symptoms of PD can be improved by drugs that replace neurotransmitters, but these treatments are unable to slow down the disease progression and often induce undesirable side effects [5]. New therapies are required to preserve DA neurons and stimulate their DA activity and to limit or halt the progression of the disease [6]. Several neurotrophins implicated in the development and maintenance of different neuronal populations have been shown to provide protection against cell death in *in vitro* and *in vivo* models of PD through diverse signaling pathways including activation of phosphatidylinositol–3-kinase (PI3-kinase)/Akt, ras-dependent mitogen-activated protein kinase (MAPK), and phospholipase C (PLC) [7]. These signaling cascades lead to the prevention of apoptotic cell death, promotion of cellular differentiation and neuritogenesis. Glial-cell derived neurotrophic factor (GDNF) has been proposed as a therapeutic agent to delay the development of PD [8]. Despite the therapeutic potential of GDNF, clinical trials have been disappointing [9], probably due to inherent drawbacks associated with the use of polypeptides applied as drugs [10], including pleiotropic effects, short half-life and inability to cross the blood-brain barrier (BBB), thus imposing repeated transcranial injections, with dramatic side effects. To obviate these issues, substantial efforts have been made to design non-peptidic small molecules with neurotrophin-like activities.

Lembehyne A (LBA), a natural polyacetylene isolated from *Haliclona sp*. marine sponges, was previously described for its neurotrophic properties for the mouse neuroblastoma cell line *Neuro2A* [11]. Structure–activity relationship studies determined the minimal structure required for activity [12]. Inspired by this natural biologically active product, we designed and synthesized an in-house collection of quinoline-derived compounds by linking the neuritogenic part of LBA to a putative neuroprotective quinoline ring largely described for its high biological potential [13]–[17] with the aim of producing chemicals possessing a neurotrophin-like activity. Herein, we report the design, synthesis and cell-based screening of small molecules exhibiting both neuroprotective and neuritogenic activities on rat mesencephalic DA neurons against spontaneous [18] or MPP^+^-induced [19] degeneration. Preliminary studies of the mechanism of action revealed that the most active compound exerts its activity through ERK1/2 signaling pathway activation, especially on DA neurons.

## Results

### Chemical syntheses

**Figure 1:**
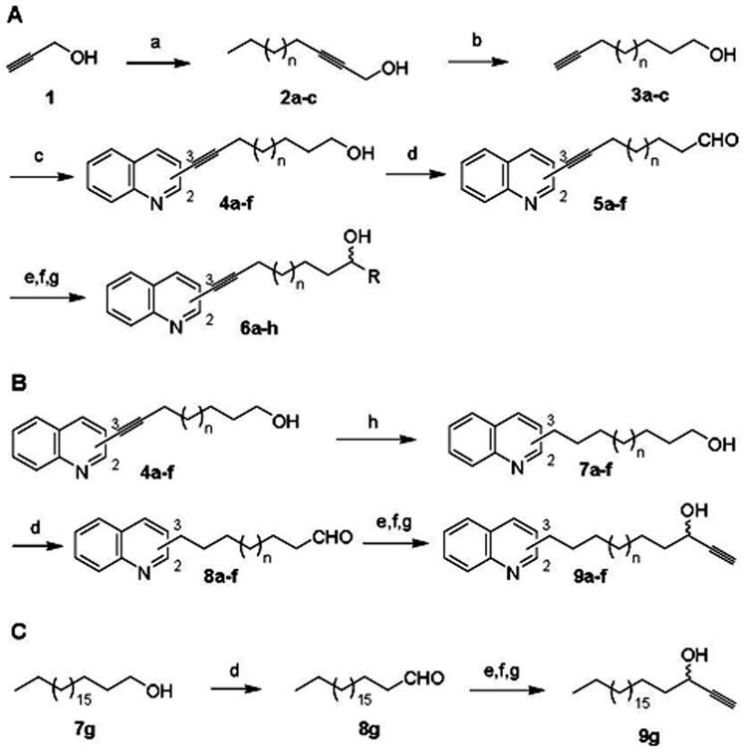
Syntheses of quinoline-derived compounds. Reagents and conditions were as follows. (A) (a) Fe(NO_3_)_2_.9H_2_O (cat), Li (3 equiv), NH_3_, −40°C to rt, 6 h; (b) NaH (6.3 equiv), NH_2_(CH_2_)_3_NH_2_, 70°C, overnight (4a) bromooctane (1 equiv) (4b) bromodecane (1 equiv) (4c) bromododecane (1 equiv); (c) PdCl_2_(PPh_3_)_2_ (0.05 equiv), CuI (0.1 equiv), Et_3_N (5 equiv), THF, reflux, 3 h. (4a–c) 2-chloroquinoline (1 equiv) (4d–f) 3-bromoquinoline (1 equiv); (d) (i) DMSO (5.8 equiv), (ClCO)_2_ (2.2 equiv), CH_2_Cl_2_, −50°C, 15 min then 2 h. (ii) Et_3_N (10 equiv), −50°C to rt; (e) (i) HC≡C-TMS (1 equiv), *n*-BuLi (1.2 equiv), −78°C, 1 h (ii) TBAF (1.06 equiv), THF, rt, 10 min. (f) BrMg- CH = CH_2_ (1.2 equiv), THF, −78°C, 2 h. (g) BrMg-cyclopropyl (1.2 equiv), THF, −78°C, 2 h. (B) (h) H_2_, Pd/C (20% w:w), EtOH_abs_, rt, overnight.

Terminal alkynols **3a–c** are the first intermediates of our multistep synthesis leading to the lateral chain of our products. First, propargylic alcohol **1** was coupled to bromoalkanes through a previously described cross-coupling reaction [20] involving an iron catalyst and lithium amide to give compounds **2a–c**. In a second step, a zipper reaction [21] led to terminal alkynols **3a–c**. This was followed by a Sonogashira cross-coupling reaction [22] between 2-chloroquinoline or 3-bromoquinoline, both commercially available, and terminal alkynols **3a–c** giving alcohol intermediates **4a–f**. Best yields were obtained by using PdCl_2_(PPh_3_)_2_ and CuI as catalysts and Et_3_N as base. Alcohol intermediates **4a–f** were then oxidized into the corresponding aldehydes through a Swern oxidation [23] to give compounds **5a–f**. This oxidation was followed by a final coupling reaction between aldehydes **5a–f** and commercially available trimethylsilylacetylene after metallation with *n*-butyllithium to give a silylated intermediate that was immediately deprotected with tetrabutylammonium fluoride leading to compounds **6a–f**. In order to study the influence of the benzylic triple bond on the biological activity, another series was synthesized from compounds **4a–f** after catalytic hydrogenation of the triple bond, leading to saturated alcohol intermediates **7a–f**. Compounds **7a–f** were then oxidized into the corresponding aldehydes **8a–f** and coupled to trimethylsilylacetylene before deprotection through the same steps as for compounds **4a–f**. With the aim of studying the influence of terminal propargylic alcohol on the biological activity, other compounds were synthesized in a similar manner from aldehyde **5c** by adding vinyl or cyclopropyl magnesium bromide to give the corresponding compounds **6g** and **6h**. Compound quinoline-free **9g** was prepared in the same way from the corresponding aldehyde **8g** of commercially available alcohol **7g**. The different steps are presented in Fig. 1. Spectroscopic data are available in the Supporting Information S1 (see *Supplementary Information - Spectroscopic analysis*).

### Screening for both Neuroprotective and Neuritogenic Activities on DA Neurons

**Figure 2:**
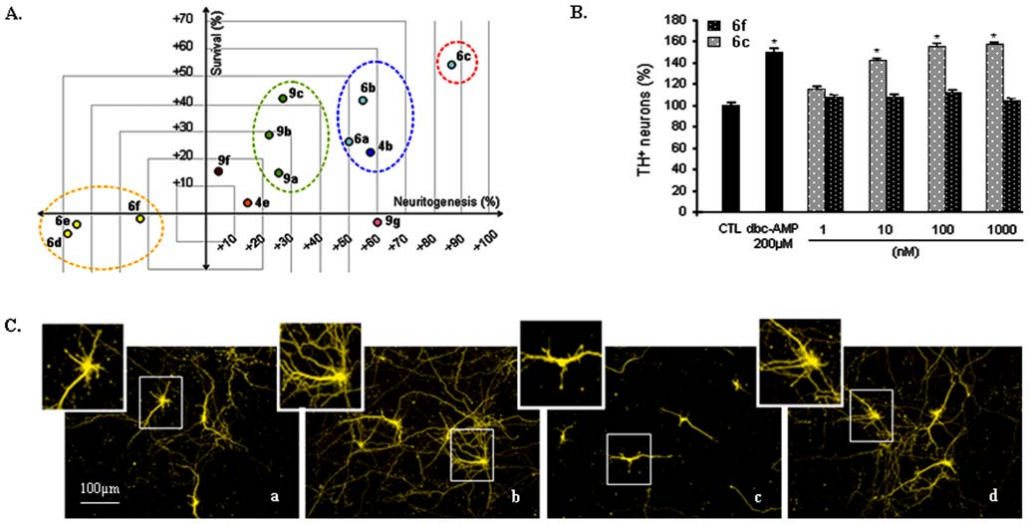
Screening for both neuroprotective and neuritogenic activity on DA neurons. (A) Two-dimensional representation of Table 1 showing four main groups of compounds exhibiting or not both neuroprotective and neuritogenic activity in different ratios. (B) The substitution position of the lateral chain is crucial for both activities (6c vs. 6f). 2-substituted compounds induced an increase in both TH neuron survival and neuritogenesis in a dose-dependent manner, while 3-substituted compounds inhibited both activities. Dbc-AMP at 200 µM was used as a positive control. Values are normalized to the non-treated control (CTL) and represent the average of at least three assays realized in triplicate and are expressed as mean±SEM. *P<0.001. Statistical analysis was performed by one-way ANOVA followed by Bonferroni's *post hoc* test. (C) Compound 6c (b) increased the number and the length of DA neuron processes compared to non-treated cultures (a). In contrast, neuritogenesis of DA neurons in compound 6f-treated cultures (c) was slowed down, showing the crucial role of the substitution position on the combined activity. (b) and (c) represent (tyrosine hydroxylase) TH immunolabeled neurons in cultures treated with 100 nM of compounds 6c and 6f. A dose-dependent neuritogenic effect was also observed for compound 6c (data not shown), while compound 6f was still inactive at higher concentrations (data not shown). Dbc-AMP at 200 µM (d) was used as a positive control. Images were acquired with an inverted fluorescent microscope coupled to a digital camera.

Synthesized compounds were then assessed for their ability to protect DA neurons from degeneration and activate neuritogenesis. The screening was performed in primary mesencephalic cultures displaying a progressive degeneration of DA neurons [18] (see *Supplementary Information - Mesencephalic cultures*). Cultures were maintained for 8 days in the presence or absence of the different tested compounds, and then tyrosine hydroxylase (TH) was immunolabeled to allow the analysis of DA neurons (see *Supplementary Information - Immunocytochemistry*). Neuroprotection was assessed by TH immunopositive (TH^+^) neuron counting (see *Supplementary Information - Survival quantification*). Neuritogenesis, expressed as total neurite length per DA neuron, was quantified using image analysis software on at least 100 neurons randomly photographed per condition (see *Supplementary Information - Neuritogenesis quantification*). Results are given in Table 1 and give rise to several interesting observations. First, compounds such as **6a–c**, **9a–c**, **4b** and **6g,h** exhibited both protective and neuritogenic activities in the nanomolar range. Second, a strong selectivity for both activities was observed between the 2- and 3-substituted quinolines (**6a–c** *vs.* **6d–f**, P<0.001, Table 1), as represented in Fig. 2B–C. Third, the activities were significantly increased with the length of the lateral chain (**6a** *vs.* **6b** *vs.* **6c**, P<0.05, Table 1) and the best dual activities were observed for compound **6c** (Table 1, Fig. 2A–C). Furthermore, saturation of the intrachain triple bond significantly decreases the activities (**6a–c** *vs.* **9a–c**, P<0.001, Table 1). Fourth, the removal (**6b** *vs.* **4b**) or replacement (**6c** *vs.* **6g**, **6c** *vs.* **6h**) of the terminal triple bond significantly decreases (P<0.001, Table 1) the protective effect but not the neuritogenic effect. The dose–effect relationship (data not shown) revealed that maximal effect was observed at a 10 nM concentration for compounds **6g** and **6h**. Finally, removal of the quinoline ring canceled out the protective effect without changing neuritogenesis (**6c** *vs.* **9g**, P<0.001, Table 1). A two-dimensional diagram showing the distribution of screened-compounds into four groups for both activities is shown in Fig. 2A. These data demonstrated a crucial role of the substitution position of the quinoline ring in this series of compounds and the contribution of both ring and terminal triple bond to the protective effect whereas the neuritogenic activity seemed to be mainly due to the presence of the lateral hydroxylated chain. Further investigations were carried out with compound **6c** which showed the best activity for both neuroprotection and neuritogenesis.

**Table 1:** Neuroprotective and neuritogenic activities of LBA-derived compounds on mesencephalic DA neurons.

R^1^-Fg-(CH_2_)_3_-(CH_2_)_n_-(CH_2_)_4_-CH(OH)R^2^					% relative to control±SEM	
Compound^a^	Functional group (Fg)	R^1^	R^2^	n	TH^+^ neurons^b^	Length/TH^+^ neuron^c^
Control					100±2.7	100±2.9
dbc-AMP					149.9±3.6	333.1±8.7
**6a**	C≡C	2-quinolinyl	C≡CH	1	127.2±7.4	150.6±7.6
**6b**	C≡C	2-quinolinyl	C≡CH	3	141.4±4.6	155.2±6.3
**6c**	C≡C	2-quinolinyl	C≡CH	5	154.5±4.1	186.0±7.7
**6d**	C≡C	3-quinolinyl	C≡CH	1	86.8±9.5	51.3±3.7
**6e**	C≡C	3-quinolinyl	C≡CH	3	89.6±2.8	55.2±2.0
**6f**	C≡C	3-quinolinyl	C≡CH	5	98.2±6.3	78.5±2.2
**9a**	CH_2_-CH_2_	2-quinolinyl	C≡CH	1	115.8±3.9	126.5±10.8
**9b**	CH_2_-CH_2_	2-quinolinyl	C≡CH	3	129.6±5.3	122.3±8.7
**9c**	CH_2_-CH_2_	2-quinolinyl	C≡CH	5	142.6±2.6	128.8±1.0
**9d**	CH_2_-CH_2_	3-quinolinyl	C≡CH	1	NR^d^	NR^d^
**9e**	CH_2_-CH_2_	3-quinolinyl	C≡CH	3	NR^d^	NR^d^
**9f**	CH_2_-CH_2_	3-quinolinyl	C≡CH	5	117.6±6.1	95.8±2.9
**4b**	C≡C	2-quinolinyl	H	3	121.4±3.1	157.9±13.9
**4e**	C≡C	3-quinolinyl	H	3	103.9±4.2	114.3±10.7
**6g**	C≡C	2-quinolinyl	◃^f^	5	135.5±2.7 e	160.6±11.4^e^
**6h**	C≡C	2-quinolinyl	CH = CH_2_	5	139.1±4.2 e	163.5±14.2^e^
**9g**	CH_2_-CH_2_	H	C≡CH	5	95.8±5.9	163.8±11.8

a Compounds used at 100 nM concentration.

b Number of TH^+^ neurons per well as a percentage of untreated cultures expressed as the mean±SEM of at least three independent experiments performed in triplicate.

c Neurite length per neuron as a percentage of untreated neurons measured on at least 100 neurons per condition using an image analyser (Neurite Outgrowth, Explora Nova, France) and expressed as the mean±SEM of three independent experiments performed in triplicate.

d Non-determined.

e Related values obtained at 10 nM because of a weak toxicity observed at 100 nM.

f ◃ = cyclopropyl. Statistical analysis was performed by one-way ANOVA followed by Bonferroni's *post hoc* test (P<0.001, *reported in text*).

### Specificity of Compound 6c-induced Neurotrophic Effect on DA Neurons

**Figure 3:**
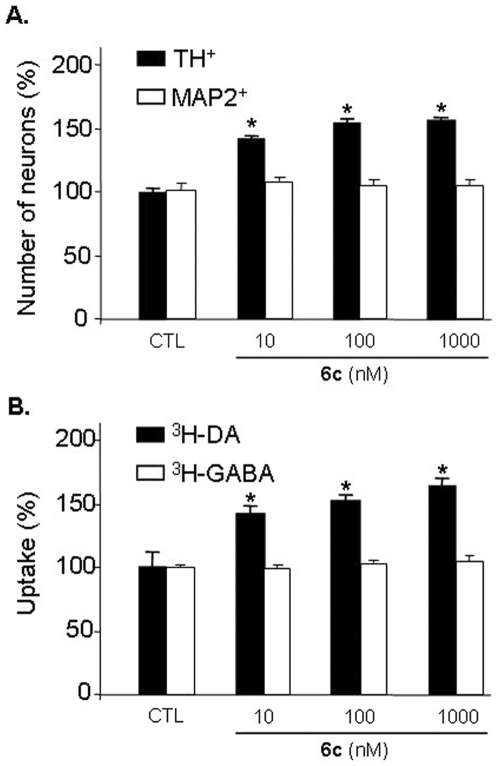
Specificity of compound 6c-induced neurotrophic effect on DA neurons. (A) The number of DA and total neurons in 6c-treated cultures was evaluated by counting of TH and MAP-2 immunolabeled neurons. The treatment with compound 6c has no effect on the viability of MAP-2^+^ neurons, while the number of TH^+^ neurons wa significantly increased in a dose-dependent manner. (B) [^3^H]-DA and [^3^H]-GABA uptake in 6c-treated cultures. Compound 6c had no effect on [^3^H]-GABA uptake, while [^3^H]-DA uptake was significantly increased in a dose-dependent manner showing the specificity of the effect of compound 6c on DA neurons in these conditions. Values are normalized to the untreated control (CTL) and represent the average of at least three assays performed in triplicate and are expressed as mean±SEM. *P<0.001. Statistical analysis was performed by one-way ANOVA followed by Bonferroni's *post hoc* test.

DA neurons represent only a few percent among the total neuronal population in ventral mesencephalon culture, which is mainly constituted by GABA (gamma-amino-*n*-butyric acid)-ergic neurons [24]. GABA neurons are not affected under these conditions so their number, assessed by counting microtubule-associated protein 2 (MAP2) immunolabeled-neurons, is only representative of total neuronal viability (Fig. 3A) [25]. To explore the phenotypic specificity of compound **6c**, uptakes of [7,8-^3^H]-DA ([^3^H]-DA) and 4-Amino-*n*-[2,3-^3^H]-butyric acid ([^3^H]-GABA) were measured under the same conditions (see *Supplementary Information – Uptake of neurotransmitters*). Uptake measurements were carried out at DIV12 to allow neuronal maturation. Fig. 3B provides evidence that DA uptake was increased in compound **6c**-treated cultures in a dose-dependent manner, while the latter has no influence on [^3^H]-GABA uptake. These data suggest that compound **6c**-induced neurotrophic activity is specific to DA neurons under these conditions.

### Protective Effect of Compound 6c Against Neurotoxin 1-methyl-4-phenylpyridinium (MPP^+^) Toxicity

**Figure 4:**
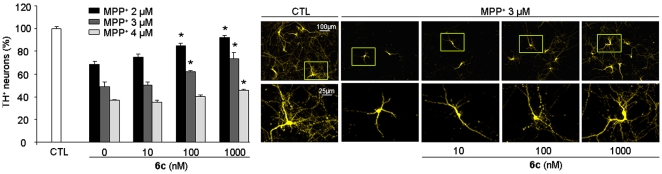
Protective effect of compound 6c against neurotoxin 1-methyl-4-phenylpyridinium (MPP^+^) toxicity. (A) Treatment of MPP^+^-treated mesencephalic cultures with compound 6c resulted in the partial rescue of DA neurons in a dose-dependent manner. Cultures were maintained from DIV1 to DIV8 in the presence or absence of compound 6c and were treated with MPP^+^ for 48 h from DIV5 to DIV7. Neuronal survival was quantified by counting TH^+^ immunolabeled neurons at DIV8. Values are normalized to a toxin-free control and represent the average of three assays performed in triplicate expressed as mean±SEM. Statistical analysis was performed by one-way ANOVA followed by Dunnett's *post hoc* test. *P<0.001. (B) Compound 6c treatment reduced MPP^+^-induced neurite degeneration. Images illustrate untreated (CTL) and treated (MPP^+^, 6c) cultures. Images were acquired with an inverted fluorescent microscope coupled to a digital camera.

Since toxins could be involved in PD onset [26]–[29], there is growing interest finding the search for compounds able to protect DA neurons from toxin-induced death. To examine more closely the protective potential of compound **6c**, MPP^+^ was added to mesencephalic cultures (*see Supplementary Information – MPP^+^ intoxication*). This inhibitor of mitochondrial complex I is specifically toxic for DA neurons in a dose-dependent manner [19]. In the present study, addition of 2, 3, or 4 µM of MPP^+^ to mesencephalic cultures between DIV5 and DIV7 decreased the number of TH^+^ neurons and induced neurite degeneration, as shown in Fig. 4A–B. Addition of compound **6c** significantly increased the survival of TH^+^ neurons at 10 nM on 2 µM MPP^+^-treated cultures (P<0.05). The effect decreased with MPP^+^ concentration but was still significant at 1 µM in 4 µM MPP^+^-treated cultures (P<0.05). In addition to rescuing TH^+^ neurons from MPP^+^-induced toxicity, compound **6c** protected neurites from MPP^+^-induced degeneration, as illustrated in Fig. 4B.

### Compound 6c-induced Neurotrophic Effect is Independent of Glial Proliferation

**Figure 5:**
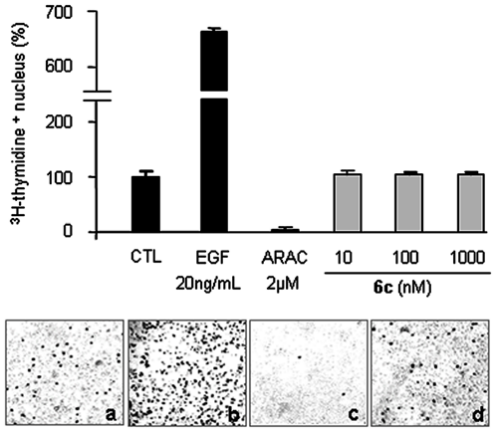
Compound 6c-induced neurotrophic effect is independent of glial proliferation. Proliferation in mesencephalic cultures was not affected by compound 6c treatment (d) compared to non-treated cultures (CTL) (a). EGF at 20 ng/mL (b) and arabinoside-C (ARA-C) at 2 µM (c), respectively, were used as controls for activation or inhibition of cell proliferation. [^3^H]-thymidine was added to the cultures for 18 h from DIV7 to DIV8. Proliferation was assessed by counting [^3^H]-thymidine^+^ nuclei appearing from autoradiographic emulsion. Images were acquired with a phase-contrast microscope coupled to a digital camera. Values are normalized to the non-treated cultures (CTL) and represent the average of three assays performed in triplicate and expressed as mean±SEM.

*In vitro* neuroprotective processes were previously linked to an inhibition or an activation of glial proliferation [30], [31]. To explore the influence of compound **6c** on proliferating cells in mesencephalic cultures, [methyl-^3^H]-thymidine, a marker of DNA synthesis used to label proliferating cells, was incorporated in the cultures for 18 hours at 37°C then washed before fixation at DIV8, as previously described [32]. An autoradiographic revelation allowed spotting and counting of radioactive nuclei. As outlined in Fig. 5, no effect of compound **6c** was observed on proliferating cells under these conditions in comparison with the mitogenic effect of epidermal growth factor (EGF) or the antimitotic effect of arabinoside-C (ARA-C). This result suggests that the compound **6c**-induced neurotrophic effect is independent of glial cell proliferation.

### Involvement of Extracellular Signal-regulated Kinase (ERK1/2) Activation in Compound 6c-induced Neurotrophic Effect

**Figure 6:**
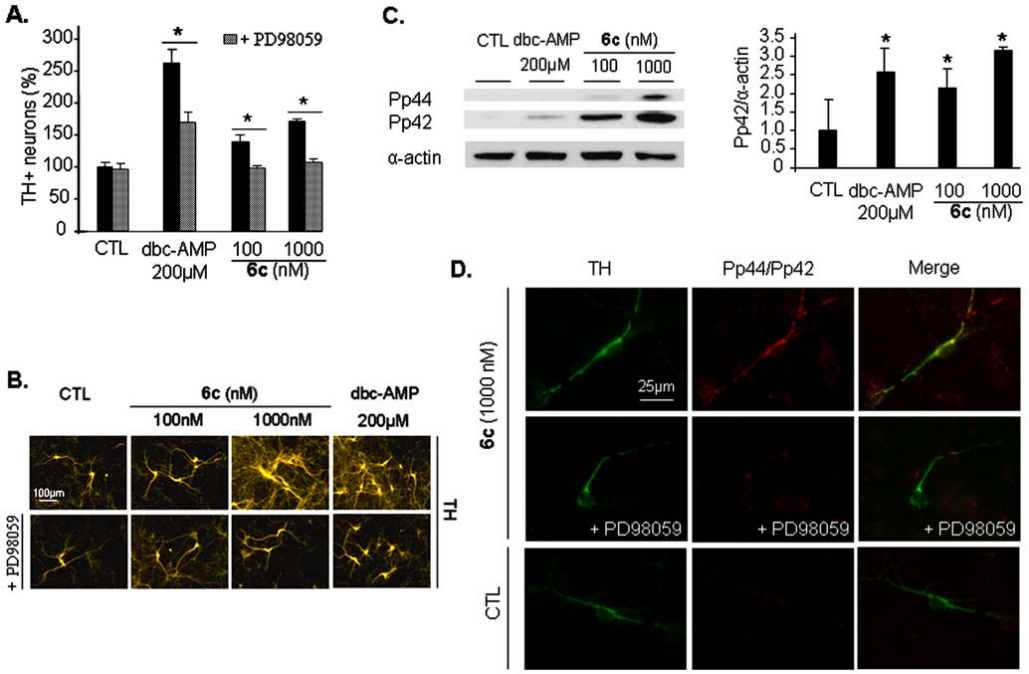
Involvement of extracellular signal-regulated kinase (ERK1/2) activation in compound 6c-induced neurotrophic effect. (A) Addition of PD98059 disrupted the compound 6c-induced neuroprotective effect. Neuronal survival was assessed by counting TH^+^ neurons at DIV12 in 100 or 1000 nM compound 6c-treated cultures in the presence or absence of the MEK inhibitor PD98059 at 10 µM. Dbc-AMP at 200 µM was used as a positive control. Values are normalized to the non-treated control and represent the average of three assays performed in triplicate and expressed as mean±SEM. Statistical analysis was performed by two-way ANOVA followed by Duncan's *post hoc* test. *P<0.05. (B) Addition of PD98059 reduced the neuritogenic effect of compound 6c. Images were acquired with an inverted fluorescent microscope coupled to a digital camera. (C) Treatment with compound 6c resulted in an increase of ERK1/2 phosphorylation in a dose-dependent manner. Western blot analyses were performed at DIV12 with proteic lysates from cultures maintained in the presence of compound 6c. Quantification of protein levels was performed with Image J software in at least three independent experiments and is expressed as mean±SEM. Statistical analysis was performed by two-way ANOVA followed by Duncan's *post hoc* test. *P<0.05. (D) Visualization of the ERK1/2 phosphorylated forms (Pp44/Pp42) in DA neurons by dual immunolabeling of TH (green) and Pp44/Pp42 (ERK1/2) (red) [48]. Treatment with compound 6c resulted in an increase of ERK1/2 phosphorylated forms in DA neurons. This effect was disrupted by the addition of PD98059 at 10 µM. Images were acquired with unchanged exposure using an inverted fluorescent microscope coupled to a digital camera. Merge were performed using image analysis software.

Activation of ERK1/2 phosphorylation has been reported to contribute to neuronal cell survival in models of neurotoxicity [33]–[35]. To further investigate the molecular signaling pathway involved in the activity induced by compound **6c**, we evaluated the effect of compound **6c** treatment upon MAPK and ERK1/2 activation (see *Supplementary Information – Western Immunoblotting of ERK1/2*). First and foremost, we proved that compound **6c** is a potent neurotrophic factor for DA neurons and further hypothesized that regulation of ERK1/2 phosphorylation might be involved in the compound **6c**-induced neurotrophic effect. First, PD98059, an inhibitor of MAPK kinase (MEK) [32], abolished the protective (Fig. 6A) and neuritogenic (Fig. 6B) effects of compound **6c**. Furthermore, stimulation of mesencephalic cultures with 100 or 1000 nM of compound **6c** resulted in an increase in ERK1/2 phosphorylation, as shown in Fig. 6C. In addition, we observed that compound **6c** treatment increased phospho-ERK1/2 immunoreactivity in DA neurons (Fig. 6D). Our results suggest that activation of the ERK1/2 pathway is an essential mechanism involved in compound **6c**-induced neurotrophic activity on DA neurons.

## Discussion

Since currently marketed CNS drugs are unable to provide a decrease in degeneration in PD [36], neuroprotective and neurorestorative approaches are ongoing [37]. In recent years, great success has been achieved on the use of neutrophins as therapeutic agents. Their potent efficacy to prevent DA neurons from death and restore DA activity was first demonstrated in cell-based and animal models [38], [39], but the following phase II clinical assays, though preliminarily encouraging, ultimately failed [40]. Given their polypeptidic structure, their large molecular weight, short half-life and inability to cross the BBB mean that surgical administration is required, with potentially severe side effects. This therefore limits their therapeutic use. Non-peptidic small molecules with neurotrophic properties may provide a mean to avoid these drawbacks [41].

We report here the discovery of quinoline-derived compounds as neurotrophin mimetics. These compounds behave as neurotrophins with nanomolar activity unprecedented for low molecular weight neurotrophic compounds. Structure–activity relationship analysis revealed not only that the presence of the quinoline ring is crucial for dual activity but also that the substitution position plays a decisive role in the neurotrophic effect. The presence of intrachain triple bond increases the activity which is in contrast with previous reports showing that unsaturations have no influence on the LBA-induced differentiation [12]. These findings suggest that the electronic delocalization between the benzylic donor moiety and the heterocyclic nitrogen in 2-substituted quinolines is strongly implicated in the effect. Furthermore, the removal or replacement of the terminal triple bond decreased the protective effect, which correlates with studies related to rasagiline [42], [43] demonstrating the potential of the propargylic group in rasagiline-induced neuroprotection. In these cases, the neuritogenic effect is not affected, which is in accordance with previous reports of compounds possessing lateral hydroxylated long chain as differentiation inducers [44]. This provides evidence that the introduction of the triple bond to the hydroxylated long chain of neuronal differentiation inducers led to compounds combining dual protective and neuritogenic activities in the nanomolar range.

All our results suggest that both the ring and the terminal propargyl alcohol contribute to the protective effect, while the neuritogenic activity seems to be mainly due to the presence of the lateral hydroxylated chain. However, this is complicated by the fact that the length of the lateral chain and the presence of the benzylic triple bond also have an influence on the protective effect.

In addition, our study demonstrates that the compound **6c**-induced neurotrophic effect on DA neurons is mediated *via* the activation of the ERK1/2 signaling pathway. This result is particularly interesting since the MAPK signaling cascade is involved in neurotrophin-induced neuronal survival and neuritogenesis [45]–[47]. The prerequisite activation of the ERK1/2 signaling pathway has also been shown to provide neuroprotection in stress-induced conditions [34], which is in line with the **6c**-induced protection observed against the oxidative stress caused by MPP^+^. These results are exciting since targeting MAPK signaling pathways represents an interesting way to slow down neurodegeneration in PD [48].

Quantitative structure–activity relationship (QSAR) studies [49], allow us to predict the potency of compound **6c** to cross the BBB by passive diffusion. Indeed, comparisons of determining factors for BBB penetration are given in Table 2. Excluding any currently unknown pharmacological parameters, the physico-chemical properties of compound **6c** are close to those expected of an orally available CNS drug, suggesting that compound **6c** is a serious candidate for *in vivo* studies and therapeutic use. Furthermore, preliminary toxically studies performed on mice show that compound **6c** does not present any toxic effect in a chronical treatment (300 mg/kg/day in a 15 days treatment) when orally administrated (results not shown).

**Table 2:** Prediction of the ability to cross the BBB of CNS drugs using QSAR_s_.

Attributes	Successful CNS Drug^a^	Compound 6c
Potent activity	low to subnanomolar	10 nM
Selectivity	High	unknown
Molecular weight	<450 g.mol^−1^	375 g.mol^−1^
logP	<5	2.04^b^
H-bond donor	<3	1
H-bond acceptor	<7	1
Rotatable bonds	<8	12
PKa	7.5–10.5 (avoid acid)	4–5 (estimated)^b^
Polar surface area (PSA)	<60–70 Å	33 Å
Aqueous solubility	>60 µg.mL^−1^	>385 µg.mL^−1c^

a see ref [49].

b see ref [50].

c in water with 1% ethanol.

In summary, we have designed and synthesized a class of quinoline-derived small molecules, some of which show potent neurotrophic activity, expressed as dual protective and trophic activities. These compounds are synthetic small molecules derived from LBA that exhibit potency in the nanomolar range. Lead compound **6c** promotes specific survival and neuritogenesis of DA neurons, preserving DA transporter activity in two relevant *in vitro* models of PD, and does not influence cellular proliferation, thus representing a potential candidate for therapeutic applications in PD.

## Materials and Methods

*More details are available in supplementary informations S1, S2, S3, S4, S5, S6, S7, S8, S9, S10*.

### Preparation and Spectroscopic Data of Compounds 2a–c-9a–g

see *Supplementary Information - S1*.

### Primary Mesencephalic Cultures

The embryos were removed at embryonic day 15.5 from pregnant Sprague-Dawley rats (Elevage Janvier, Le Genest St. Isle, France) that had been anesthetized, then decerebrated. Ventral mesencephalon were dissected and collected. Cell suspensions prepared by mechanical trituration precoated overnight with 1 mg/ml polyethylenimine in borate buffer, pH 8.3. The cells were then maintained for maturation and differentiation in B27-supplemented Neurobasal culture medium. Cultures were treated at DIV1 and every four days 300 µL of culture medium were replaced by medium supplemented with treatments. For more details See *Supplementary Information - S2*.

### Tyrosine Hydroxylase (TH) Immunolabeling

After 12 min fixation with a 4% formaldehyde solution in Dulbecco's phosphate-buffered saline (PBS), cells were washed three times with PBS and then incubated in PBS^+^ (PBS containing 0.2% Triton X-100, 10% fetal bovine serum (Sigma, Saint Louis, MO) and 0.01% thimerozal (Sigma, Saint Quentin Fallavier, France) for 1 hour. The cells were further incubated overnight at 4°C with a rabbit anti-TH polyclonal antibody or a mouse anti-MAP-2 monoclonal antibody. Subsequent incubations were performed, at room temperature, with a secondary anti-rabbit IgG cyanin 3 conjugate or an Alexa Fluor 488 F(ab')2 fragment of goat anti-rabbit IgG or an anti-mouse IGg cyanin 3 conjugate. Concerning phospho-ErK1/2 (pp42/pp44) immunofluorescence staining, the cultures were incubated overnight at 4°C with a mouse monoclonal anti phospho-ERK1/2 antibody diluted at 1∶100 in PBS^+^ then washed and incubated with an anti-mouse IGg cyanin 3 conjugate. For more details See *Supplementary Information - S3*.

### Neuroprotection was Assessed by TH Immunopositive (TH^+^) Neuron counting

For more details See *Supplementary Information - S4*.

### Neuritogenesis, Quantification

For more details See *Supplementary Information - S5*.

### MPP^+^ Intoxication of Mesencephalic Cultures

MPP^+^ intoxication was performed according to a method previously described [19]. For more details See *Supplementary Information – S6*.

### Uptakes of [7.8-^3^H]-DA ([^3^H]-DA) and 4-Amino-*n*-[2,3-^3^H]butyric acid ([^3^H]-GABA)

High-affinity uptake of [^3^H]-DA and [^3^H]-GABA was determined according to a method previously described [25]. For more details See *Supplementary Information – S7*.

### Uptakes of [méthyl-^3^H]-Thymidine

Mesencephalic treated cultures with 10, 100 or 1000 nM of compound **6c** were exposed to 1 µCi of [methyl-^3^H]-thymidine After

washes, the cells were fixed and positive nuclei were visualized with emulsion then quantified by counting. For more details See *Supplementary Information – S8*.

### ERK1/2 Activation

ERK1/2 activation was assessed according to previously described methods [47]. For more details See *Supplementary Information – S9*.

### Statistical Analysis

Comparisons between two groups were performed with Student's *t* test. Multiple comparisons against a single reference group were made by one-way analysis of variance (ANOVA) followed by Dunnett's or Bonferroni's *post-hoc* tests. When all pairwise comparisons were made, two-way ANOVA was used followed by Duncan's test. S.E.M. values were derived from at least three values per condition of three independent experiments.

## Supporting Information

Supporting Information S1 (.15 MB DOC)
