# Supplementary material for: Chemicals Possessing a Neurotrophin-Like Activity on Dopaminergic Neurons in Primary Culture
Source: PLoS One. 2009 Jul 10;4(7):e6215. doi: 10.1371/journal.pone.0006215 (PMC2704893; doi:10.1371/journal.pone.0006215)
Supplement: Supporting Information S1 — (.15 MB DOC) [file pone.0006215.s001.doc]

**SUPPPORTING INFORMATION**

**S1: Spectroscopic analysis** Tetrahydrofuran was distilled over sodium/ benzophenone under nitrogen prior to use. Dichloromethane, DMSO and triethylamine were distilled over calcium hydride. Bromoalkanes were distilled before use. All reactions involving moisture-sensitive reactants were performed under nitrogen atmosphere using oven-dried glassware. Routine monitoring of reactions was performed using Riedel-deHaën, S, 0.063 mm, 0.032 silica gel plates, which were dipped in a sulfomolybdic acid solution or o-vanillin in EtOH/H_2_SO_4_ (95:5) and then heated to allow revelation or dipped few min in a mixture of silica and iodide. Flash chromatography was performed using Riedel-deHaën, S, 0.063 mm, 0.032 silica under moderate pressure with the appropriate solvent giving us a migrate front (Rf) in the range of 0.2 to 0.3. ^1^H NMR spectra were recorded on BRUKER AR 200 P (200 MHz) and AM-400 (400 MHz) spectrometers as solutions in CDCl_3_. Chemical shifts are expressed in parts per million (ppm, δ) downfield from tetramethylsilane (TMS) and are referenced to CHCl_3_ (7.26 ppm) as internal standard. ^13^C NMR spectra were recorded on BRUKER AR 200 P (200 MHz) and AM-400 (100 MHz) spectrometers as solutions in CDCl_3_. Chemical shifts are expressed in parts per million (ppm, δ) downfield from tetramethylsilane (TMS) and are referenced to CHCl_3_ (77.0 ppm) as internal standard. The attribution of the different carbons (C, CH, CH_2_, CH_3_) was determined by ^13^C to ^1^H polarization transfer (J modulation). Infrared spectra (IR, cm^-1^) were measured on a BRUKER vector 22 FT spectrometer. Mass spectra (MS) were measured on a Nermag-Sidar R10-10C spectrometer with a quadripolar filter.

**Undec-2-yn-1-ol** (**2a**). Ammonia (100mL) was condensed at −35°C then lithium (2.5 mmol, 0.05 equiv) was added leading to a blue solution. A catalytic quantity of Fe(NO_3_)_2_ and lithium (150 mmol, 3 equiv) was introduced and the mixture was stirred for 10 min. Propargylic alcohol (75 mmol, 1.5 equiv) dissolved in THF (40mL) was added dropwise over 15 min and bromooctane (50 mmol, 1 equiv) dissolved in THF (40mL) was also added over 15 min. The mixture was stirred for 6 hours and then ammonia was slowly evaporated overnight. The solution was stopped by adding water (100 mL) and extracted three times with dichloromethane (3 x 200 mL). The combined organic layers were dried over MgSO_4_ then filtered and concentrated *in vacuo.* The compound was then purified by flash chromatography with a mixture of cyclohexane and ethyl acetate (80: 20) to give clear yellow oil. Yield: 60% (7.62 g). ^1^H NMR (200 MHz): δ 0.88 (t, *J*= 6.8 Hz, 3H), 1.41 (m, 12H), 2.21 (tt, *J*= 6.8 Hz, *J*= 2.3 Hz, 2H), 4.24 (t, *J*= 2.2 Hz, 2H). ^13^C NMR (50 MHz): δ 14.0, 18.5, 18.7, 22.6, 28.6, 28.8, 29.1, 29.2, 31.8, 51.4. IR (cm^-1^): ν 3332, 2925, 2855, 2236.

**Tridec-2-yn-1-ol** (**2b**). Yield: 75% (11.03 g). ^1^H NMR (200 MHz): δ 0.88 (t, *J* = 6.8 Hz, 3H), 1.41 (m, 16H), 2.20 (tt, *J* = 6.8 Hz, *J* = 2.2 Hz, 2H), 4.25 (t, *J* = 2.2 Hz, 2H). ^13^C NMR (50 MHz): δ 14.1, 18.7, 22.6, 28.6, 28.8, 29.1, 29.3, 29.5, 29.6, 31.9, 51.4. IR (cm^-1^): ν 3335, 2922, 2853, 2228.

**Pentadec-2-yn-1-ol** (**2c**). Yield: 58% (9.61 g). ^1^H NMR (200 MHz): δ 0.88 (t, *J* = 6.2 Hz, 3H), 1.41 (m, 20H), 2.20 (tt, *J* = 6.8 Hz, *J* = 2.0 Hz, 2H), 4.25 (t, *J* = 2.2 Hz, 2H). ^13^C NMR (50 MHz): δ 14.0, 18.5, 18.7, 22.6, 28.6, 28.8, 29.1, 29.2, 31.8, 51.4. IR (cm^-1^): ν 3332, 2925, 2855, 2236.

**Undec-10-yn-1-ol** (**3a**). NaH 60% (283.5 mmol, 6.3 equiv.) was introduced under nitrogen and washed three times with pentane.1,3-diaminopropane (990 mmol, 22 equiv.) distilled from calcium hydride was then added dropwise. The mixture was heated at 70°C for one hour. Compound **2a** (45 mmol, 1 equiv) was added and the solution was heated at 70°C for 2 hours then 50°C overnight. The mixture was slowly hydrolyzed by adding water (100 mL) then extracted three times with ethyl acetate (3 x 200 mL). The combined organic layers were washed three times with an HCl 0.1 M aqueous solution (3 x 100 mL) then dried over MgSO_4_, filtered and concentrated *in vacuo.* The compound was then purified by flash chromatography with a mixture of cyclohexane and ethyl acetate (90: 10) to give clear yellow wax. Yield: 75% (4.28 g). ^1^H NMR (200 MHz): 1.44 (m, 14H), 1.93 (t, *J* = 2.4 Hz, 1H), 2.17 (td, *J* = 6.8 Hz, *J* = 2.4 Hz, 2H), 3.63 (t, *J* = 6.4 Hz, 2H). ^13^C NMR (50 MHz): δ 18.3, 25.6, 28.4, 28.6, 28.9, 29.4, 32.7, 62.9, 67.9, 84.7. IR (cm^-1^): ν 3330, 3310, 2926, 2855, 2117.

**Tridec-12-yn-1-ol** (**3b**). Yield: 67% (5.88 g). ^1^H NMR (200 MHz): δ 1.44 (m, 18H), 1.91 (t, *J* = 2.6 Hz, 1H), 2.14 (td, *J* = 6.8 Hz, *J* = 2.6 Hz, 2H), 3.61 (t, *J* = 6.6, 2H). ^13^C NMR (50 MHz): δ 18.3, 25.7, 28.4, 28.7, 29.0, 29.4, 32.7, 62.9, 67.9, 84.7. IR (cm^-1^): ν 3330, 3310, 2926, 2855, 2117.

**Pentadec-14-yn-1-ol** (**3c**). Yield: 97% (9.8 g). ^1^H NMR (200 MHz): δ 1.44 (m, 22H), 1.91 (t, *J*= 2.8 Hz, 1H), 2.16 (td, *J*= 2.6 Hz, *J*= 7.0 Hz, 2H), 3.61 (t, *J*= 6.6 Hz, 2H). ^13^C NMR (50 MHz): δ 18.3, 25.7, 28.4, 28.5, 28.7, 29.1, 29.4, 32.7, 62.9, 67.9, 84.7. IR (cm^-1^): ν 557, 631, 668, 749, 1050, 1215, 1465, 2856, 2928, 3015, 3308.

**11-(quinolin-2-yl)undec-10-yn-1-ol (4a).** To a solution of compound **3a** (4.5 mmol, 1 equiv) and 2-chloroquinoline (5.85 mmol, 1.3 equiv) in triethylamine (22.5 mmol, 5 equiv) and THF (10 mL) under nitrogen were added PdCl_2_(PPh_3_)_2_ (0.23 mmol, 0.05 equiv) and CuI (0.45 mmol, 0.1 equiv). The mixture was heated at 70°C for 3 hours, then quenched by adding water (10 mL) and extracted three times with dichloromethane (3 x 100 mL). The combined organic layers were washed three times with an HCl 0.1 M aqueous solution (3 x 50 mL) then dried over MgSO_4_, filtered and concentrated *in vacuo*. The compound was then purified by flash chromatography with a mixture of cyclohexane and ethyl acetate (70: 30). Yield: 87% (1.21 g). ^1^H NMR (400 MHz): δ 1.26 (m, 16H + 1 H_2_O, 2H), 1.42 (m, 2H), 1.49 (m, 2H), 1.61 (m, 2H), 2.48 (t, *J*= 7.2 Hz, 2H), 3.62 (t, *J*= 6.8 Hz, 2H), 7.44 (d, *J*= 8 .4 Hz, 1H), 7.49 (t, *J*= 7.2 Hz, 1H), 7.68 (t, *J*= 7.6 Hz, 1H), 7.75 (d, *J*= 8.4 Hz, 1H), 8.06 (d, *J*= 8.4 Hz, 2H). ^13^C NMR (100 MHz): δ 19.4, 25.7, 28.3, 28.9, 29.0, 29.2, 29.3, 29.4, 29.5, 29.6, 29.7, 31.2, 32.7, 38.1, 62.9, 76.6, 73.3, 81.0, 92.2, 124.1, 126.6, 126.8, 127.3, 129.1, 129.7, 135.9, 144.1, 148.0. IR (cm^-1^): ν 617, 637, 694, 721, 754, 787, 829, 871, 953, 1057, 1120, 1141, 1238, 1261, 1277, 1307, 1336, 1373, 1424, 1463, 1500, 1555, 1595, 1617, 2226, 2853, 2925, 3058, 3312. EI-MS-m/z: 295 (M**^+^**, 2), 278 (6), 264 (13), 250 (17), 236 (34), 222 (80), 208 (76), 194 (54), 180 (100), 166 (42), 140 (38), 128 (26).

**13-(quinolin-2-yl)tridec-12-yn-1-ol (4b).** Yield: 76% (1.13 g). ^1^H NMR (400 MHz): δ 1.28 (s, 12H + 1 H_2_O, 2H), 1.49 (m, 2H), 1.55 (m, 2H), 1.66 (m, 2H), 2.48 (t, *J*= 7.2 Hz, 2H), 3.62 (t, *J*= 6.4 Hz, 2H), 7.43 (d, *J*= 8.4 Hz, 1H), 7.49 (t, *J*= 7.6 Hz, 1H), 7.68 (t, *J*= 8.0 Hz, 1H), 7.75 (d, *J*= 8.4 Hz, 1H), 8.05 (d, *J*= 8.4 Hz, 1H). ^13^C NMR (100 MHz): δ 19.4, 25.6, 26.8, 28.9, 29.1, 29.3, 29.4, 29.5, 30.1, 32.7, 43.4, 62.9, 81.0, 92.1, 124.1, 126.6, 126.8, 127.3, 129.1, 129.8, 135.9, 144.1, 147.9. IR (cm^-1^): ν 617, 638, 665, 679, 722, 753, 786, 829, 870, 952, 1056, 1120, 1142, 1237, 1261, 1277, 1307, 1373, 1424, 1463, 1500, 1555, 1595, 1617, 2226, 2852, 2923, 3337. EI-MS-m/z: 323 (M**^+^**, 1), 306 (2), 292 (3), 278 (6), 264 (11), 250 (17), 236 (35), 222 (70), 208 (56), 194 (44), 180 (100), 166 (29), 140 (26), 128 (16). ESI-HRMS Calcd for [M+H]^+^ C_22_H_30_NO: 324.2327. Found: 324.2330.

**15-(quinolin-2-yl)pentadec-14-yn-1-ol (4c).** Yield: 44% (2.07 g). ^1^H NMR (400 MHz): δ 1.32 (s, 8H + 1 H_2_O, 2H), 1.46 (m, 2H), 1.55 (m, 2H), 1.64 (m, 2H), 2.48 (t, *J*= 7.2 Hz, 2H), 3.62 (t, *J*= 6.8 Hz, 2H), 7.43 (d, *J*= 8.4 Hz, 1H), 7.49 (t, *J*= 7.6 Hz, 1H), 7.68 (t, *J*= 8.0 Hz, 1H), 7.75 (d, *J*= 8.4 Hz, 1H), 8.06 (d, *J*= 8.4 Hz, 2H). ^13^C NMR (50 MHz): δ 19.4, 25.6, 28.2, 28.8, 28.9, 29.2, 29.3, 32.7, 62.8, 81.1, 92.1, 124.1, 126.6, 126.8, 127.3, 129.0, 129.7, 135.8, 144.0, 147.9. IR (cm^-1^): ν 617, 639, 664, 679, 723, 753, 786, 829, 871, 953, 1056, 1120, 1141, 1238, 1261, 1277, 1307, 1336, 1373, 1424, 1462, 1500, 1555, 1595, 1617, 2226, 1854, 2926, 3059, 3337. EI-MS-m/z: 351 (M^+^, 6), 334 (4), 320 (3), 306 (2), 292 (5), 278 (12), 264 (15), 250 (20), 236 (42), 222 (78), 208 (67), 194 (51), 180 (100), 166 (35), 140 (22), 128 (15). ESI-MS m/z: 352 ([M+H]**^+^**, 100). Anal. Calcd for C_24_H_33_NO, 4.2 H_2_0: C, 81.01, H, 9.48, N, 3.94. Found: C, 81.01, H, 9.11, N, 3.72. ESI-HRMS Calcd for [M+H]^+^ C_24_H_34_NO: 352.2640. Found: 352.2632.

**11-(quinolin-3-yl)undec-10-yn-1-ol (4d).** To a solution of compound **3d** (10 mmol, 1 equiv) and 3-bromoquinoline (13 mmol, 1.3 equiv) in triethylamine (50 mmol, 5 equiv) and THF (20 mL) under nitrogen were added PdCl_2_(PPh_3_)_2_ (0.5 mmol, 0.05 equiv.) and CuI (1 mmol, 0.1 equiv). The mixture was heated at 70°C for 3 hours, then quenched by adding water (20 mL) and extracted three times with dichloromethane (3 x 200 mL). The combined organic layers were washed three times with an HCl 0.1 M aqueous solution (3 x 200 mL) then dried over MgSO_4_, filtered and concentrated *in vacuo*. The compound was then purified by flash chromatography with a mixture of cyclohexane and ethyl acetate (70: 30). Yield: 65% (1.93 g). ^1^H NMR (400 MHz): δ 1.32 (s, 8H), 1.47 (t, *J*= 7Hz, 2H), 1.57 (m, 2H), 1.62 (m, 2H), 2.45 (t, *J*= 7.2 Hz, 2H), 3.62 (t, *J*= 6.8 Hz, 2H), 7.51 (t, *J*= 7.6 Hz, 1H), 7.66 (t, *J*= 7.6 Hz, 1H), 7.72 (d, *J*= 8.0 Hz, 1H), 8.05 (d, *J*= 8.4 Hz, 1H), 8.14 (s, 1H), 8.85 (s, 1H). ^13^C NMR (100 MHz): δ 19.3, 25.7, 28.4, 28.7, 28.8, 29.2, 29.2, 32.6, 62.4, 77.7, 94.0, 118.1, 126.9, 127.2, 128.9, 129.4, 137.8, 146.1, 152.2. IR (cm^-1^): ν 605, 665, 750, 784, 861, 908, 956, 1056, 1126, 1215, 1346, 1464, 1489, 1568, 2230, 2853, 2924, 3343. EI-MS-m/z: 295 (M^+^, 12), 278 (5), 264 (7), 250 (10), 236 (21), 222 (45), 208 (50), 194 (90), 180 (100), 166 (68), 140 (26).

**13-(quinolin-3-yl)tridec-12-yn-1-ol (4e).** Yield: 63% (2.02 g). ^1^H NMR (400 MHz): δ 1.29 (s, 12H), 1.49 (m, 2H), 1.53 (m, 2H), 1.64 (m, 2H), 2.46 (t, *J*= 7.2 Hz, 2H), 3.62 (t, *J*= 6.8 Hz, 2H), 7.52 (t, *J*= 8.0 Hz, 1H), 7.67 (t, *J*= 8.0 Hz, 1H), 7.73 (d, *J*= 8.4 Hz, 1H), 8.06 (d, *J*= 8.4 Hz, 1H), 8.15 (s, 1H), 8,85 (s, 1H). ^13^C NMR (100 MHz): δ 19.5, 25.7, 26.8, 28.5, 28.8, 29.0, 29.2, 29.4, 29.5, 30.1, 32.8, 43.4, 62.8, 77.8, 94.2, 118.2, 127.1, 127.3, 129.1, 129.6, 137.9, 146.3, 152.3. IR (cm^-1^): ν 613, 644, 719, 752, 784, 862, 879, 908, 928, 953, 974, 1006, 1038, 1056, 1110, 1132, 1190, 1349, 1416, 1469, 1489, 1568, 1620, 2015, 2232, 2358, 2849, 2916, 3310. EI-MS-m/z: 323 (M**^+^**, 19), 306 (7), 292 (10), 278 (14), 264 (18), 250 (25), 236 (45), 222 (79), 208 (75), 194 (89), 180 (100), 166 (82), 140 (46). ESI-HRMS Calcd for [M+H]^+^ C_22_H_30_NO: 324.2327. Found: 324.2321.

**15-(quinolin-3-yl)pentadec-14-yn-1-ol (4f).** Yield: 56% (1.84 g). ^1^H NMR (400 MHz): δ 1.32 (s, 16H), 1.48 (m, 2H), 1.55 (m, 2H), 1.63 (m, 2H), 2.46 (t, *J*= 7.2 Hz, 2H), 3.62 (t, *J*= 6.4 Hz, 2H), 7.51 (t, *J*= 8.0 Hz, 1H), 7.66 (t, *J*= 6.8 Hz, 2H), 7.72 (d, *J*= 8.4 Hz, 1H), 8.05 (d, *J*= 8.4 Hz, 1H), 8.14 (s, 1H), 8,85 (s, 1H). ^13^C NMR (100 MHz): δ 19.4, 25.7, 28.5, 28.8, 29.0, 29.5, 32.7, 62.8, 77.8, 94.1, 118.2, 127.0, 127.3, 129.1, 129.5, 137.8, 146.3, 152.4. IR (cm^-1^): ν 606, 644, 723, 751, 784, 861, 908, 957, 1056, 1127, 1283, 1346, 1412, 1463, 1489, 1570, 1710, 2231, 2835, 2859, 2933, 3342. EI-MS-m/z: 351 (M^+^, 5), 292 (1), 278 (3), 264 (4), 250 (5), 236 (11), 222 (22), 208 (20), 194 (36), 180 (73), 166 (44), 140 (14).

**11-(quinolin-2-yl)undec-10-yn-1-al (5a).** To a solution of oxalyl chloride (0.77 mmol, 2.2 equiv) in dried dichloromethane (5 mL) under nitrogen at –60°C, DMSO (2.03 mmol, 5.8 equiv) was added dropwise. After 15 min, a solution of compound **4a** (0.35 mmol, 1 equiv) in dichloromethane (5 mL) was added. The temperature was maintained between –60°C and –50°C for 2 hours then triethylamine (3.5 mmol, 10 equiv) was added. The mixture was stirred overnight then quenched by adding water (10 mL) and extracted three times with dichloromethane (3 x 100 mL). The combined organic layers were washed three times with water (3 x 200 mL) and then with an HCl 0.1 M aqueous solution (3 x 200 mL), then dried over MgSO_4_, filtered and concentrated *in vacuo*. The compound was then purified by flash chromatography in a mixture of cyclohexane and ethyl acetate (90: 10). Yield: 68% (70 mg). ^1^H NMR (400 MHz): δ 1.50 (m, 12H + 1 H_2_O, 2H), 2.40 (t, *J*= 6.8 Hz, 2H), 2.48 (t, *J*= 6.8 Hz, 2H), 7.45 (d, *J*= 8.4 Hz, 1H), 7.52 (t, *J*= 7.6 Hz, 1H), 7.68 (t, *J*= 7.2 Hz, 1H), 7.76 (d, *J*= 8.0 Hz, 1H), 8.06 (d, *J*= 8.4 Hz, 2H), 9.75 (s, 1H). ^13^C NMR (100 MHz): δ 19.4, 21 .9, 28.1, 28.2, 28.6, 28.8, 28.9, 29.0, 29.1, 29.2, 29.6, 43.7, 81.0, 91.9, 123.9, 124.1, 126.6, 126.8, 127.3, 127.4, 129.0, 129.7, 135.9, 143.9, 147.9, 149.5, 202.7. IR (cm^-1^): ν 625, 668, 745, 831, 909, 1215, 1425, 1463, 1501, 1555, 1596, 1722, 2227, 2856, 2929.

**13-(quinolin-2-yl)tridec-12-yn-1-al (5b).** Yield: 49% (156 mg). ^1^H NMR (300 MHz): δ 1.50 (m, 16H + 1 H_2_O, 2H), 2.40 (td, *J*= 1.8 Hz, *J*= 7.2 Hz, 2H), 2.49 (t, *J*= 7.2 Hz, 2H), 7.44 (dd, *J*= 1.5, *J*= 8.4 Hz, 1H), 7.49 (t, *J*= 7.2 Hz, 1H), 7.68 (td, *J*= 1.5 Hz, *J*= 8.4 Hz, 1H), 7.75 (d, *J*= 8.4 Hz, 1H), 8.06 (d, *J*= 8.4 Hz, 2H), 9.75 (t, *J*= 1.5 Hz, 1H). ^13^C NMR (75 MHz): δ 19.4, 22.0, 26.8, 28.3, 28.9, 29.1, 29.3, 29.4, 29.6, 30.1, 43.8, 81.1, 92.0, 124.1, 126.6, 126.9, 127.3, 129.1, 129.7, 135.7, 144.1, 148.0, 202.7. IR (cm^-1^): ν 585, 669, 751, 909, 1215, 1464, 1723, 2850, 2918.

**15-(quinolin-2-yl)pentadec-14-yn-1-al (5c).** Yield: 82% (286 mg). ^1^H NMR (200 MHz): δ 1.50 (m, 20H), 2.39 (td, *J*= 2.0 Hz, *J*= 7,4 Hz, 2H), 2.49 (t, *J*= 7.0 Hz, 2H), 7.45 (d, *J*= 8.4 Hz, 1H), 7.50 (t, *J*= 7.4 Hz, 1H), 7.67 (dd, *J*= 1.2 Hz, *J*= 8.4 Hz, 1H), 7.75 (d, *J*= 8.4 Hz, 1H), 8.06 (d, *J*= 8.2 Hz, 2H), 9.74 (t, *J*= 1.6 Hz, 1H). ^13^C NMR (50 MHz): δ 19.5, 22.0, 24.7, 28.3, 28.9, 29.1, 29.4, 34.1, 43.8, 92.5, 124.2, 126.7, 126.9, 127.3, 128.9, 129.0, 129.8, 136.0, 144.0, 147.9, 193.7. IR (cm^-1^): ν 557, 622, 649, 727, 831, 905, 1045, 1123, 1142, 1239, 1374, 1425, 1464, 1501, 1556, 1595, 1618, 1711, 2227, 2853, 2924.

**11-(quinolin-3-yl)undec-10-yn-1-al (5d).** Yield: 95% (290 mg). ^1^H NMR (200 MHz): δ 1.38 (m, 12H + 1 H_2_O, 2H), 2.39 (tt, *J*= 1.0 Hz, *J*= 7.4 Hz, 2H), 2.45 (t, *J*= 6.8 Hz, 2H), 7.50 (t, *J*= 7.8 Hz, 1H), 7.64 (dd, *J*= 1.0 Hz, *J*= 7.0 Hz, 1H), 7.70 (t, *J*= 8.2 Hz, 1H), 8.06 (d, *J*= 8.4 Hz, 1H), 8.13 (d, *J*= 1.0 Hz, 1H), 8.85 (d, *J*= 2.0 Hz, 1H), 9.73 (t, *J*= 1.0 Hz, 1H). ^13^C NMR (50 MHz): δ 19.4, 21.9, 24.8, 28.3, 28.4, 28.7, 28.8, 29.0, 29.1, 29.1, 34.1, 43.7, 77.8, 94.1, 94.2, 118.5, 127.1, 127.3, 128.8, 129.6, 138.1, 146.0, 146.1, 152.2, 177.2, 202.6. IR (cm^-1^): ν 571, 607, 666, 751, 784, 861, 909, 957, 1127, 1214, 1347, 1412, 1463, 1491, 1569, 1720, 2230, 2853, 2923.

**13-(quinolin-3-yl)tridec-12-yn-1-al (5e).** Yield: 63% (170 mg). ^1^H NMR (400 MHz): δ 1.38 (m, 16H), 2.35 (t, *J*= 7.6 Hz, 2H), 2.46 (t, *J*= 6.8 Hz, 2H), 7.54 (t, *J*= 7.6 Hz, 1H), 7.68 (t, *J*= 7.2 Hz, 1H), 7.75 (d, *J*= 8.0 Hz, 1H), 8.09 (d, *J*= 8.4 Hz, 1H), 8.17 (s, 1H), 8.88 (d, *J*= 1.2 Hz, 1H), 9.75 (s, 1H). ^13^C NMR (50 MHz): δ 19.5, 22.1, 24.7, 28.5, 28.5, 28.7, 29.1, 29.0, 29.3, 29.4, 33.9, 77.7, 100.6, 127.2, 127.4, 128.7, 128.9, 129.8, 130.0, 138.2, 152.2, 170.6. IR (cm^-1^): ν 566, 607, 665, 723, 752, 784, 862, 909, 958, 1130, 1188, 1346, 1412, 1463, 1492, 1573, 1620, 1710, 2231, 2853, 2924.

**15-(quinolin-3-yl)pentadec-14-yn-1-al (5f).** Yield: 84% (248 mg). ^1^H NMR (400 MHz): δ 1.40 (m, 14H), 1.6 (m, 6H), 2.39 (td, *J*= 2.0 Hz, *J*= 7.4 Hz, 2H), 2.47 (t, *J*= 7.0 Hz, 2H), 7.53 (td, *J*= 1.2 Hz, *J*= 8.0 Hz, 1H), 7.68 (td, *J*= 1.6 Hz, *J*= 8.4 Hz, 1H), 7.71 (t, *J*= 8.2 Hz, 1H),  8.06 (d, *J*= 8.6 Hz, 1H), 8.14 (d, *J*= 1.8 Hz, 1H), 8.86 (d, *J*= 2.0 Hz, 1H), 9.75 (t, *J*= 1.8 Hz, 1H). ^13^C NMR (50 MHz): δ 19.5, 22.1, 26.9, 28.6, 28.9, 29.1, 29.3, 29.4, 29.5, 30.2, 43.8, 94.1, 101.2, 118.3, 126.9, 127, 127.2, 127.3, 129.1, 129.3, 129.5, 137.8, 146.5, 152.5, 202.9. IR (cm^-1^): ν 607, 628, 668, 748, 861, 909, 1215, 1347, 1464, 1489, 1723, 2854, 2926.

**13-(quinolin-2-yl)trideca-1,12-diyn-3-ol (6a).** To a solution of trimethylsilylacetylene (0.072 mmol, 1 equiv) in THF (5 mL) under nitrogen *n*-Butyllithium (2.5 M, 0.072 mmol, 1 equiv) was added dropwise. The mixture was stirred for one hour at –78°C and kept cold. To a solution of aldehyde **5a** (0.072 mmol, 1 equiv) in THF (5mL) under nitrogen at –78 °C, freshly prepared organolithium solution (0.086 mmol, 1.2 equiv) was added dropwise. The mixture was stirred at –78°C for 2 hours then quenched by adding water (5 mL) and extracted three times with dichloromethane (3 x 20 mL). The combined organic layers were washed three times with water (3 x 60 mL) and then with an HCl 0.1 M aqueous solution (3 x 60 mL), then dried over MgSO_4_, filtered and concentrated *in vacuo*. The residue was dissolved in THF (8 mL) and stirred under nitrogen at room temperature in presence of TBAF (1M) (0.076 mmol, 1.06 equiv). After 10 min’ reaction the mixture was quenched by adding water (8 mL) and extracted three times with ethyl acetate (3 x 30 mL). The combined organic layers were washed three times with successive saturated solutions of NaHCO_3_ (3 x 100 mL) and NaCl (3 x 100 mL), dried over MgSO_4_, filtered and concentrated *in vacuo*. The compound was then purified by flash chromatography with a mixture of cyclohexane and ethyl acetate (90: 10). Yield: 72% (23 mg). ^1^H NMR (400 MHz): δ 1.25 (m, 10H + 1 H_2_O, 2H), 1.45 (m, 2H), 1.69 (m, 2H), 2.44 (d, *J*= 2.0 Hz, 1H), 2.49 (t, *J*= 7.2 Hz, 2H), 4.37 (td, *J*= 2.0 Hz, *J*= 6.4 Hz, 1H), 7.45 (d, *J*= 8.4 Hz, 1H), 7.51 (t, *J*= 7.2 Hz, 1H), 7.69 (td, *J*= 1.2 Hz, *J*= 6.8 Hz, 1H), 7.76 (d, *J*= 8.0 Hz, 1H), 8.07 (d, *J*= 8.4 Hz, 2H). ^13^C NMR (50 MHz): δ 19.5, 25.0, 28.3, 29.0, 29.1, 29.2, 29.4, 29.5, 37.7, 62.4, 72.7, 81.1, 92.2, 115.1, 124.2, 126.7, 126.9, 127.4, 129.2, 129.8, 134.4, 136.0, 136.1, 148.1. IR (cm^-1^): ν 626, 752, 786, 830, 871, 953, 1033, 1121, 1141, 1238, 1261, 1277, 1308, 1374, 1425, 1463, 1501, 1556, 1595, 1618, 2226, 2853, 2924, 3306. ESI-MS m/z: 320 ([M+H]**^+^**, 100). ESI-HRMS Calcd for [M+H]^+^ C_22_H_26_NO: 320.2014. Found: 320.2017.

**15-(quinolin-2-yl)pentadeca-1,14-diyn-3-ol (6b).** Yield: 92% (53 mg). ^1^H NMR (400 MHz): δ 1.25 (m, 14H + 1 H_2_O, 2H), 1.50 (m, 2H), 1.70 (m, 2H), 2.44 (d, *J*= 2.0 Hz, 1H), 2.49 (t, *J*= 7.2 Hz, 2H), 4.37 (td, *J*= 2.0 Hz, *J*= 6.4 Hz, 1H), 7.45 (d, *J*= 8.4 Hz, 1H), 7.51 (t, *J*= 7.6 Hz, 1H), 7.69 (td, *J*= 1.2 Hz, *J*= 6.8 Hz, 1H), 7.76 (d, *J*= 8.0 Hz, 1H), 8.07 (d, *J*= 8.2 Hz, 2H). ^13^C NMR (50 MHz): δ 19.5, 25.0, 28.3, 29.0, 29.1, 29.2, 29.4, 29.5, 37.7, 62.4, 72.7, 81.1, 92.2, 115.1, 124.2, 126.7, 126.9, 127.4, 129.2, 129.8, 134.4, 136.0, 136.1, 148.1. IR (cm^-1^): ν 570, 627, 665, 751, 830, 870, 1036, 1073, 1121, 1216, 1238, 1262, 1277, 1376, 1425, 1463, 1501, 1556, 1596, 1618, 1722, 2227, 2363, 2854, 2925, 3307. ESI-MS m/z: 348 ([M+H]**^+^**, 100). ESI-HRMS Calcd for [M+Na]^+^ C_24_H_29_NO: 370.2147. Found: 370.2148.

**17-(quinolin-2-yl)heptadeca-1,16-diyn-3-ol (6c).** Yield: 74% (47 mg). ^1^H NMR (400 MHz): δ 1.25 (m, 18H + 1 H_2_O, 2H), 1.48 (m, 2H), 1.67 (m, 2H), 2.44 (d, *J*= 2.0 Hz, 1H), 2.50 (t, *J*= 7.2 Hz, 2H), 4.38 (td, *J*= 1.6 Hz, *J*= 6.8 Hz, 1H), 7.45 (d, *J*= 8.4 Hz, 1H), 7.52 (t, *J*= 7.6 Hz, 1H), 7.70 (t, *J*= 7.2 Hz, 1H), 7.77 (d, *J*= 8.0 Hz, 1H), 8.08 (d, *J*= 8.0 Hz, 2H). ^13^C NMR (50 MHz): δ 19.5, 28.2, 28.8, 28.9, 29.0, 29.1, 29.2, 29.3, 37.6, 62.2, 122.3, 124.2, 126.8, 127.4, 129.0, 130.0, 136.1, 44.1, 148.0. IR (cm^-1^): ν 572, 627, 668, 746, 831, 929, 1049, 1214, 1425, 1501, 1596, 2231, 2361, 2401, 2856, 2928, 3019. ESI-MS m/z: 376 ([M+H]**^+^**, 100). Anal. Calcd for C_26_H_33_NO,2.4 H_2_0: C, 81.53, H, 8.90, N, 3.66. Found: C, 81.51, H, 8.60, N, 3.46. ESI-HRMS Calcd for [M+H]^+^ C_26_H_36_NO: 376.2640. Found: 376.2646.

**13-(quinolin-3-yl)trideca-1,12-diyn-3-ol (6d).** Yield: 61% (66 mg). ^1^H NMR (400 MHz): δ 1.28 (m, 6H), 1.46 (m, 4H), 1.65 (m, 4H), 2.44 (d, *J*= 2.4 Hz, 1H), 2.47 (t, *J*= 6.8 Hz, 2H), 4.37 (td, *J*= 2.0 Hz, *J*= 6.8 Hz, 1H), 7.53 (t, *J*= 8.0 Hz, 1H), 7.68 (td, *J*= 1.2 Hz, *J*= 8.4 Hz, 1H), 7.74 (d, *J*= 8.0 Hz, 1H), 8.08 (d, *J*= 8.4 Hz, 1H), 8.16 (s, 1H), 8.86 (d, *J*=1.6 Hz, 1H). ^13^C NMR (50 MHz): δ 19.5, 25.0, 28.5, 28.9, 29.0, 29.2, 29.2, 29.3, 29.5, 29.5, 37.7, 62.2, 72.6, 77.9, 83.9, 94.2, 118.3, 127.1, 127.4, 129.1, 129.7, 138.0, 146.3, 152.4. IR (cm^-1^): ν 559, 629, 722, 752, 784, 862, 909, 957, 1024, 1079, 1128, 1346, 1413, 1464, 1491, 1570, 1597, 1619, 1713, 2232, 2363, 2852, 2923, 3306. ESI-MS m/z: 320 ([M+H]**^+^**, 100). ESI-HRMS Calcd for [M+H]^+^ C_22_H_26_NO: 320.2014. Found: 320.2008.

**15-(quinolin-3-yl)pentadeca-1,14-diyn-3-ol (6e).** Yield: 42% (25 mg). ^1^H NMR (400 MHz): δ 1.25 (m, 10H + 3 H_2_O, 6H), 1.48 (m, 4H), 1.70 (m, 4H), 2.44 (d, *J*= 2.0 Hz, 1H), 2.48 (t, *J*= 6.8 Hz, 2H), 4.38 (td, *J*= 1.6 Hz, *J*= 6.4 Hz, 1H), 7.54 (t, *J*= 8.0 Hz, 1H), 7.69 (td, *J*= 1.6 Hz, *J*= 7.2 Hz, 1H), 7.75 (d, *J*= 8.0 Hz, 1H), 8.08 (d, *J*= 8.4 Hz, 1H), 8.17 (s, 1H), 8.88 (s, 1H). ^13^C NMR (50 MHz): δ 19.5, 25.0, 28.5, 28.5, 28.8, 28.9, 29.1, 29.3, 29.5, 29.6, 37.7, 62.2, 72.7, 94.3, 127.2, 127.4, 128.2, 129.1, 129.7, 138.0, 139.5, 152.4, 158.4. IR (cm^-1^): ν 561, 569, 581, 653, 669, 754, 785, 909, 1067, 1261, 1347, 1464, 1491, 1570, 1716, 2010, 2233, 2339, 2360, 2859, 2926, 3303. ESI-MS m/z: 348 ([M+H]**^+^**, 100). ESI-HRMS Calcd for [M+H]^+^ C_24_H_30_NO: 348.2327. Found: 348.2324.

**17-(quinolin-3-yl)heptadeca-1,16-diyn-3-ol (6f).** Yield: 30% (15 mg). ^1^H NMR (400 MHz): δ 1.25 (m, 14H + 3H_2_O, 6H), 1.48 (m, 4H), 1.65 (m, 4H), 2.45 (d, *J*= 2.4 Hz, 1H), 2.47 (t, *J*= 7.2 Hz, 2H), 4.38 (td, *J*= 2.1 Hz, *J*= 6.6 Hz, 1H), 7.54 (t, *J*= 7.5 Hz, 1H), 7.69 (td, *J*= 1.2 Hz, *J*= 8.4 Hz, 1H), 7.75 (d, *J*= 8.1 Hz, 1H), 8.07 (d, *J*= 8.4 Hz, 1H), 8.16 (d, *J*=1.5 Hz, 1H), 8.87 (d, *J*= 1.5 Hz, 1H). ^13^C NMR (50 MHz): δ 19.5, 25.0, 26.9, 28.5, 28.9, 29.0, 29.2, 29.3, 33.9, 37.7, 62.3, 72.8, 85.0, 94.2, 118.3,127.1, 127.4, 129.1, 129.7, 135.7, 138.0, 146.3, 152.4. IR (cm^-1^): ν 608, 665, 750, 784, 862, 910, 958, 1025, 1130, 1215, 1346, 1413, 1463, 1491, 1571, 1709, 2231, 2360, 2855, 2928, 3302. ESI-MS m/z: 376 ([M+H]**^+^**, 100). ESI-HRMS Calcd for [M+H]^+^ C_26_H_34_NO: 376.2640. Found: 376.2656.

**15-(quinolin-2-yl)pentadec-1-cyclopropyl-14-yn-1-ol (6g).** To a solution of aldehyde **5c** (0.2 mmol, 1 equiv.) in THF (5mL) under nitrogen at –78 °C were added 0.24 mmol of a commercially available solution of cyclopropylmagnesium bromide (1.2 equiv). The mixture was stirred at –78°C for 2 hours then was quenched by adding water (10 mL) and extracted three times with dichloromethane (3 x 50 mL). The combined organic layers were dried over MgSO_4_, filtered and concentrated *in vacuo*. The compound was then purified by flash chromatography with a mixture of cyclohexane and ethyl acetate (80: 20). Yield: 41% (32 mg). ^1^H NMR (300 MHz): δ 0.22 (m, 2H), 0.49 (m, 2H), 0.88 (m, 1H), 1.27 (m, 14H), 1.49 (m, 4H), 1.63 (m, 5H), 2.49 (t, *J*= 7.2 Hz, 2H), 2.84 (tdd, *J*= 2.0 Hz, *J*= 3.0 Hz, *J*= 6.0 Hz, 1H), 7.45 (d, 8.4 Hz, 1H), 7.50 (t, 7.8 Hz, 1H), 7.69 (t, 7.8 Hz, 1H), 7.76 (d, 8.4 Hz, 1H), 8.07 (d, 8.4 Hz, 2H). ^13^C NMR (50 MHz): δ 2.4, 2.7, 18.0, 19.5, 25.7, 26.9, 28.3, 29.0, 29.1, 29.6, 29.7, 37.2, 81.0, 92.2, 124.2, 126.7, 126.9, 127.4, 129.1, 129.8, 135.9, 144.1, 148.0. ESI-MS m/z: 392 ([M+H]**^+^**, 100). Anal. Calcd for C_27_H_37_NO, 1.1 H_2_0: C, 79.49, H, 9.59, N, 3.43. Found: C, 79.55, H, 9.23, N, 3.28. ESI-HRMS Calcd for [M+H]^+^ C_27_H_37_NO: 392.2953. Found: 392.2948.

**17-(quinolin-2-yl)heptadec-1-ene-16-yn-3-ol (6h).** To a solution of aldehyde **5c** (0.2 mmol, 1 equiv.) in THF (5mL) under nitrogen at –78 °C were added 0.24 mmol of a commercially available solution of vinylmagnesium bromide (1.2 equiv). The mixture was stirred at –78°C for 2 hours, then was quenched by adding water (10 mL) and extracted three times with dichloromethane (3 x 50 mL). The combined organic layers were dried over MgSO_4_, filtered and concentrated *in vacuo*. The compound was then purified by flash chromatography with a mixture of cyclohexane and ethyl acetate (80: 20) Yield: 81% (61 mg). ^1^H NMR (300 MHz): δ 1.27 (m, 14H), 1.50 (m, 5H), 1.67 (m, 3H), 2.49 (t, *J*= 7.2 Hz, 2H), 4.09 (q, 6.0 Hz, 1H), 5.09 (d, *J*= 10.5 Hz, 1H), 5.21 (d, J= *J*= 17.1 Hz, 1H), 5.86 (td, 6.3 Hz, 3.9 Hz, 1H), 7.47 (d, 8.4 Hz, 1H), 7.51 (t, 7.2 Hz, 1H), 7.70 (t, 7.8 Hz, 1H), 7.77 (d, 8.1 Hz, 1H), 8.08 (d, 8.4 Hz, 2H). ^13^C NMR (50 MHz): δ 25.3, 26.9, 28.3, 29.0, 29.1, 29.5, 29.5, 37.0, 73.3, 77.2, 114.5, 124.2, 124.7, 125.9, 126.7, 127.4, 129.1, 129.9, 136.0, 141.3. ESI-MS m/z: 378 ([M+H]**^+^**, 100). Anal. Calcd for C_26_H_35_NO, 1.4 H_2_0: C, 79.99, H, 9.40, N, 3.53. Found: C, 80.07, H, 8.99, N, 3.49. ESI-HRMS Calcd for [M+H]^+^ C_26_H_36_NO: 378.2797. Found: 378.2804.

**11-(quinolin-2-yl)undecan-1-ol (7a).** To a solution of compound **4a** (1 mmol, 1 equiv) in absolute ethanol (3 mL) under nitrogen, Pd/C (40mg, ~20% w/w) was added. Nitrogen was then replaced by hydrogen and the mixture was stirred overnight at room temperature, then quenched by dropwise addition of water (1 mL), filtered through celite then washed with ethyl acetate (3 x 20 mL). The organic layer was dried over MgSO_4_ then concentrated *in vacuo.* Yield: 99% (298 mg). ^1^H NMR (400 MHz): δ 1.26 (m, 14H), 1.55 (m, 2H), 1.80 (m, 2H), 2.96 (t, *J*= 7.8 Hz, 2H), 3.63 (t, *J*= 6.6 Hz, 2H), 7.29 (t, *J*= 8.4 Hz, 1H), 7.47 (t, *J*= 8.1 Hz 1H), 7.67 (td, *J*= 1.5 Hz, *J*= 7.2 Hz, 1H), 7.76 (d, *J*= 7.8 Hz, 1H), 8.05 (m, 2H). ^13^C NMR (100 MHz): δ 25.7, 29.4, 29.5, 29.5, 29.6, 30.1, 32.8, 39.3, 63.0, 77.2, 121.3, 125.6, 127.4, 128.7, 129.3, 136.2, 147.8, 163.1. IR (cm^-1^): ν 623, 672, 725, 753, 775, 790, 834, 866, 894, 949, 974, 1013, 1054, 1124, 1210, 1321, 1381, 1427, 1461, 1504, 1567, 1601, 1619, 2848, 2919, 3055, 3253.

**13-(quinolin-2-yl)tridecan-1-ol (7b).** Yield: 99% (515 mg). ^1^H NMR (400 MHz): δ 1.25 (m, 18H), 1.54 (m, 3H), 1.80 (m, 2H), 2.96 (t, *J*= 7.8 Hz, 2H), 3.64 (t, *J*= 6.6 Hz, 2H), 7.30 (d, *J*= 8.4 Hz, 1H), 7.48 (t, *J*= 7.2 Hz, 1H), 7.68 (td, *J*= 1.5 Hz, *J*= 6.9 Hz, 1H), 7.77 (d, *J*= 8.1 Hz, 1H), 8.05 (m, 2H). ^13^C NMR (100 MHz): δ 25.7, 29.4, 29.5, 30.1, 32.8, 39.4, 63.1, 77.2, 121.4, 125.6, 127.5, 128.8, 129.3, 136.2, 163.1. IR (cm^-1^): ν 623, 672, 725, 754, 777, 791, 835, 867, 903, 964, 1057, 1124, 1217, 1261, 1322, 1380, 1427, 1462, 1505, 1567, 1601, 1619, 2848, 2917, 3054, 3262. ESI-MS-m/z: 328 ([M+H]**^+^**, 100). ESI-HRMS Calcd for [M+H]^+^ C_22_H_34_NO: 328.2640. Found: 328.2642.

**15-(quinolin-2-yl)pentadecan-1-ol (7c).** Yield: 99% (627 mg). ^1^H NMR (400 MHz): δ 1.25 (m, 22H), 1.56 (m, 2H), 1.81 (m, 2H), 2.97 (t, *J*= 7.8 Hz, 2H), 3.64 (t, *J*= 6.6 Hz, 2H), 7.30 (d, *J*= 8.7 Hz, 1H), 7.48 (t, *J*= 7.8 Hz, 1H), 7.68 (td, *J*= 1.5 Hz, *J*= 6.3 Hz, 1H), 7.78 (d, *J*= 7.8 Hz, 1H), 8.05 (m, 2H). ^13^C NMR (100 MHz): δ 25.7, 29.5, 30.1, 32.8, 39.3, 63.1, 121.4, 125.6, 126.7, 127.5, 128.7, 129.3, 126.3, 147.8, 163.1. IR (cm^-1^): ν 623, 672, 725, 744, 777, 791, 835, 867, 903, 964, 1057, 1124, 1215, 1261, 1322, 1380, 1427, 1462, 1505, 1567, 1601, 1619, 2325, 2848, 2917, 3054, 3263. ESI-MS-m/z: 356 ([M+H]**^+^**, 100).

**13-(quinolin-3-yl)tridecan-1-ol (7e).** Yield: 99% (162 mg). ^1^H NMR (400 MHz): δ 1.25 (m, 14H + 3 H_2_O, 6H), 1.56 (m, 2H), 1.71 (m, 2H), 2.78 (t, *J*= 7.5 Hz, 2H), 3.63 (t, *J*= 6.6 Hz, 2H), 7.51 (t, *J*= 7.2 Hz, 1H), 7.65 (td, *J*= 1.2 Hz, *J*= 6.9 Hz 1H), 7.75 (d, *J*= 8.1 Hz, 1H), 7.91 (d, *J*= 1.2 Hz, 1H), 8.07 (d, *J*= 8.4 Hz, 1H), 8,76 (d, *J*= 1.5 Hz, 1H). ^13^C NMR (100 MHz): δ 25.8, 27.2, 29.2, 29.4, 29.6, 31.1, 32.6 32.8, 33.2, 63.0, 126.5, 127.3, 128.2, 128.5, 129.0, 130.4, 134.2, 135.4, 146.7, 152.1. IR (cm^-1^): ν 613, 652, 718, 750, 788, 864, 908, 961, 988, 1057, 1124, 1349, 1471, 1495, 1573, 2849, 2916, 3282. ESI-MS-m/z: 328 ([M+H]**^+^**, 100). ESI-HRMS Calcd for [M+H]^+^ C_22_H_34_NO: 328.2640. Found: 328.2650.

**15-(quinolin-3-yl)pentadecan-1-ol (7f).** Yield: 99% (704 mg). ^1^H NMR (400 MHz): δ 1.25 (m, 22H), 1.54 (m, 2H), 1.71 (m, 2H), 2.79 (t, *J*= 7.2 Hz, 2H), 3.63 (t, *J*= 6.6 Hz, 2H), 7.51 (t, *J*= 7.2 Hz, 1H), 7.65 (t, *J*= 7.0 Hz, 1H), 7.76 (d, *J*= 8.1 Hz, 1H), 7.91 (d, *J*= 1.2 Hz, 1H), 8.08 (d, *J*= 8.4 Hz, 1H), 8,77 (s, 1H). ^13^C NMR (100 MHz): δ 25.8, 27.2, 29.2, 29.4, 29.6, 31.1, 32.8, 33.2, 63.0, 126.6, 127.3, 128.2, 128.6, 134.3, 135.4, 146.6, 152.0. IR (cm^-1^): ν 614, 633, 718, 751, 788, 864, 907, 961, 983, 1009, 1056, 1076, 1136, 1195, 1354, 1471, 1495, 1573, 2849, 2916, 3273. ESI-MS-m/z: 356 ([M+H]**^+^**, 100).

**11-(quinolin-2-yl)undecan-1-al (8a).** To a solution of oxalylchloride (0.59 mmol, 2.2 equiv.) in dried dichloromethane (5 mL) under nitrogen at –60°C, DMSO (1.56 mmol, 5.8 equiv) was added dropwise. After 15 min, a solution of compound **4a** (0.27 mmol, 1 equiv) in dichloromethane (5 mL) was added. The temperature was maintained between –60°C and –50°C for 2 hours, then triethylamine (2.7 mmol, 10 equiv) was added. The mixture was stirred overnight then quenched by adding water (10 mL) and extracted three times with dichloromethane (3 x 100 mL). The combined organic layers were washed three times with water (3 x 200 mL) and then with an HCl 0.1 M aqueous solution (3 x 200 mL), then dried over MgSO_4_, filtered and concentrated *in vacuo*. The compound was then purified by flash chromatography with a mixture of cyclohexane and ethyl acetate (90: 10). Yield: 50% (40 mg). ^1^H NMR (400 MHz): δ 1.27 (m, 12H + 1 H_2_O, 2H), 1.65 (m, 2H), 1.77 (m, 2H), 2.39 (td, *J*= 1.8 Hz, *J*= 7.8 Hz, 2H), 2.97 (t, *J*= 7.8 Hz, 2H), 7.29 (d, *J*= 8.4 Hz, 1H), 7.47 (t, *J*= 7.2 Hz 1H), 7.67 (td, *J*= 1.5 Hz, *J*= 6.9 Hz, 1H), 7.76 (d, *J*= 8.1 Hz, 1H), 8.05 (d, *J*= 8.4 Hz, 1H), 9.74 (s, 1H). ^13^C NMR (50 MHz): δ 22.0, 28.6, 28.9, 29.1, 29.3, 29.5, 29.5, 298.7, 30.0, 30.1, 39.3, 43.9, 77.3, 121.4, 125.7, 128.3, 128.7, 136.6, 147.8, 163.1, 203.0. IR (cm^-1^): ν 621, 665, 723, 756, 828, 870, 954, 1017, 1122, 1142, 1185, 1310, 1427, 1464, 1505, 1564, 1601, 1619, 1720, 2852, 2923.

**13-(quinolin-2-yl)tridecan-1-al (8b).** Yield: 91% (180 mg). ^1^H NMR (400 MHz): δ 1.25 (m, 16H + 1 H_2_O, 2H), 1.64 (m, 2H), 1.80 (m, 2H), 2.40 (td, *J*= 1.8 Hz, *J*= 7.2 Hz, 2H), 2.96 (t, *J*= 7.8 Hz, 2H), 7.28 (d, *J*= 8.4 Hz, 1H), 7.47 (t, *J*= 7.2 Hz 1H), 7.67 (td, *J*= 1.5 Hz, *J*= 6.9 Hz, 1H), 7.77 (d, *J*= 8.1 Hz, 1H), 8.04 (t, *J*= 8.4 Hz, 1H), 9.75 (t, *J*= 2.1 Hz, 1H). ^13^C NMR (100 MHz): δ 22.0, 26.9, 29.1, 29.3, 29.4, 29.5, 29.5, 30.0, 39.4, 43.9, 77.2, 121.3, 125.6, 126.7, 127.4, 128.8, 129.3, 136.2, 147.8, 163.1, 203.0. IR (cm^-1^): ν 578, 619, 722, 756, 827, 955, 1017, 1117, 1142, 1184, 1310, 1427, 1464, 1505, 1563, 1601, 1619, 1722, 2852, 2922.

**15-(quinolin-2-yl)pentadecan-1-al (8c).** Yield: 88% (264 mg). ^1^H NMR (400 MHz): δ 1.20 (m, 20H), 1.57 (m, 2H), 1.85 (m, 2H), 2.37 (td, *J*= 1.8 Hz, *J*= 7.2 Hz, 2H), 3.30 (t, *J*= 7.8 Hz, 2H), 7.53 (d, *J*= 8.7 Hz, 1H), 7.67 (t, *J*= 7.2 Hz 1H), 7.86 (t, *J*= 7.2 Hz, 1H), 7.96 (d, *J*= 8.4 Hz, 1H), 8.67 (d, *J*= 8.1 Hz, 1H), 8.70 (d, *J*= 8.4 Hz, 1H), 9.72 (t, *J*= 1.8 Hz, 1H). ^13^C NMR (100 MHz): δ 22.0, 28.6, 28.9, 29.1, 29.3, 29.5, 29.5, 298.7, 30.0, 30.1, 39.3, 43.9, 77.3, 121.4, 125.7, 128.3, 128.7, 136.6, 147.8, 163.1, 203.0. IR (cm^-1^): ν 578, 619, 722, 756, 827, 955, 1017, 1117, 1142, 1184, 1310, 1427, 1464, 1505, 1563, 1601, 1619, 1722, 2852, 2922.

**13-(quinolin-3-yl)tridecan-1-al (8e).** Yield: 66% (196 mg). ^1^H NMR (400 MHz): δ 1.25 (m, 16H + 4 H_2_O, 8H), 1.64 (m, 2H), 1.71 (m, 2H), 2.41 (td, *J*= 1.8 Hz, *J*= 7.2 Hz, 2H), 2.79 (t, *J*= 7.5 Hz, 2H), 7.52 (t, *J*= 7.8 Hz, 1H), 7.66 (t, *J*= 7.2 Hz, 1H), 7.77 (d, *J*= 7.8 Hz, 1H), 7.93 (s, 1H), 8.09 (d, *J*= 8.4 Hz, 1H), 8.78 (s, 1H), 9.75 (t, *J*= 1.8 Hz, 1H). ^13^C NMR (100 MHz): δ 22.0, 26.9, 29.1, 29.3, 29.4, 29.5, 29.6, 30.1, 31.1, 33.2, 43.9, 77.2, 126.6, 127.3, 128.6, 128.8, 134.3, 146.5, 151.8, 203.0. IR (cm^-1^): ν 558, 615, 648, 722, 751, 786, 861, 907, 958, 1018, 1129, 1197, 1464, 1498, 1578, 1720, 2852, 2922.

**15-(quinolin-3-yl)pentadecan-1-al (8f).** Yield: 70% (237 mg). ^1^H NMR (400 MHz): δ 1.25 (m, 20H + 2 H_2_O, 4H), 1.64 (m, 2H), 1.71 (m, 2H), 2.41 (td, *J*= 1.8 Hz, *J*= 7.5 Hz, 2H), 2.79 (t, *J*= 7.5 Hz, 2H), 7.51 (t, *J*= 7.2 Hz, 1H), 7.65 (td, *J*= 1.5 Hz, *J*= 6.9 Hz, 1H), 7.76 (d, *J*= 8.1 Hz, 1H), 7.91 (s, 1H), 8.07 (d, *J*= 8.4 Hz, 1H), 8.77 (s, 1H), 9.74 (s, 1H). ^13^C NMR (100 MHz): δ 22.1, 29.1, 29.3, 29.4, 29.6, 29.6, 31.1, 32.2, 43.9, 77.2, 126.5, 127.3, 128.5, 129.1, 134.1, 146.7, 152.1, 180.2, 203.0. IR (cm^-1^): ν 565, 614, 648, 722, 750, 787, 860, 905, 956, 983, 1017, 1125, 1264, 1330, 1464, 1495, 1571, 1606, 1724, 2852, 2922.

**Octadecanal (8g).** Yield: 99% (999 mg). ^1^H NMR (300 MHz): δ 0.84 (t, *J*= 6.0 Hz, 3H), 1.23 (s, 28H), 1.59 (t, *J*= 6.9 Hz, 2H), 2.38 (t, *J*= 7.5 Hz, 2H), 9.73 (s, 1H). ^13^C NMR (100 MHz): δ 14.0, 22.0, 22.6, 29.3, 29.6, 29.5, 31.8, 43.9, 202.7.

**13-(quinolin-2-yl)tridec-1-yn-3-ol (9a).** To a solution of trimethylsilylacetylene (0.018 mmol, 1 equiv) in THF (5 mL) under nitrogen, *n*-Butyllithium (2.5M, 0.018 mmol, 1 equiv) was added dropwise. The mixture was stirred for one hour at –78°C and kept cold. To a solution of aldehyde **5a** (0.018 mmol, 1 equiv) in THF (5mL) under nitrogen at –78 °C, freshly prepared organolithium solution (0.021 mmol, 1.2 equiv) was added dropwise. The mixture was stirred at –78°C for 2 hours then quenched by adding water (5 mL) and extracted three times with dichloromethane (3 x 20 mL). The combined organic layers were washed three times with water (3 x 60 mL) and then with an HCl 0.1 M aqueous solution (3 x 60 mL), then dried over MgSO_4_, filtered and concentrated *in vacuo*. The residue was dissolved in THF (8 mL) and stirred under nitrogen at room temperature in presence of TBAF (0.190 mmol, 1.06 equiv). After 10 min reaction the mixture was quenched by adding water (8 mL) and extracted three times with ethyl acetate (3 x 30 mL). The combined organic layers were washed three times with successive saturated solutions of NaHCO_3_ (3 x 100 mL) and NaCl (3 x 100 mL), dried over MgSO_4_, filtered and concentrated *in vacuo*. The compound was then purified by flash chromatography with a mixture of cyclohexane and ethyl acetate (90: 10). Yield: 84% (5 mg). ^1^H NMR (300 MHz): δ 1.28 (m, 14H), 1.70 (m, 2H), 1.82 (m, 2H), 2.44 (d, *J*= 2.1 Hz, 1H), 2.98 (t, *J*= 7.8 Hz, 2H), 4.37 (td, J= 2.1 Hz, *J*= 6.6 Hz, 1H), 7.30 (d, *J*= 8.4 Hz, 1H), 7.47 (t, *J*= 7.8 Hz, 1H), 7.68 (td, *J*= 1.5 Hz, *J*= 6.9 Hz, 1H), 7.77 (d, *J*= 8.1 Hz, 1H), 8.07 (d, *J*= 8.4 Hz, 1H). ^13^C NMR (50 MHz): δ 25.0, 29.1, 29.2, 29.3, 29.5, 31.0, 33.1, 37.7, 61.9, 72.3, 126.5, 127.2, 128.1, 128.6, 128.8, 134.3, 135.3, 146.4, 151.8. IR (cm^-1^): ν 648, 721, 751, 787, 861, 907, 957, 986, 1047, 1128, 1330, 1379, 1465, 1498, 1575, 1716, 2021, 2160, 2341, 2360, 2852, 2922, 3308. ESI-MS m/z: 324 ([M+H]**^+^**, 100). ESI-HRMS Calcd for [M+H]^+^ C_22_H_30_NO: 324.2327. Found: 324.2324.

**15-(quinolin-2-yl)pentadec-1-yn-3-ol (9b).** Yield: 50% (60 mg). ^1^H NMR (400 MHz): δ 1.25 (m, 18H), 1.70 (m, 2H), 1.82 (m, 2H), 2.44 (d, *J*= 2.1 Hz, 1H), 2.97 (t, *J*= 7.8 Hz, 2H), 4.38 (td, J= 2.1 Hz, *J*= 6.6 Hz, 1H), 7.30 (d, *J*= 8.4 Hz, 1H), 7.47 (t, *J*= 7.8 Hz, 1H), 7.67 (td, *J*= 1.5 Hz, *J*= 6.9 Hz, 1H), 7.76 (d, *J*= 8.1 Hz, 1H), 8.06 (d, *J*= 8.4 Hz, 1H). ^13^C NMR (50 MHz): δ 25.0, 29.2, 29.4, 29.5, 30.1, 37.7, 39.2, 62.2, 72.2, 85.2, 121.4, 125.6, 126.7, 127.4, 128.6, 129.3, 136.3, 147.7, 163.1. IR (cm^-1^): ν 557, 621, 649, 722, 752, 786, 861, 907, 957, 986, 1019, 1129, 1232, 1265, 1330, 1464, 1498, 1577, 1713, 2852, 2922, 3308. ESI-MS m/z: 352 ([M+H]**^+^**, 100). ESI-HRMS Calcd for [M+H]^+^ C_24_H_34_NO: 352.2640. Found: 352.2636.

**17-(quinolin-2-yl)heptadec-1-yn-3-ol (9c).** Yield: 50% (57 mg). ^1^H NMR (300 MHz): δ 1.26 (m, 18H), 1.75 (m, 4H), 2.97 (t, *J*= 7.5 Hz, 1H), 4.37 (td, J= 2.1 Hz, *J*= 6.6 Hz, 1H), 7.30 (d, *J*= 8.4 Hz, 1H), 7.48 (td, *J*= 0.9 Hz, *J*= 8.1 Hz, 1H), 7.68 (td, *J*= 1.5 Hz, *J*= 6.9 Hz, 1H), 7.77 (d, *J*= 8.1 Hz, 1H), 8.07 (d, *J*= 8.4 Hz, 1H). ^13^C NMR (50 MHz): δ 25.0, 26.9, 29.2, 29.5, 29.5, 30.0, 37.7, 39.3, 62.2, 72.7, 85.2, 121.4, 125.7, 126.7, 127.4, 128.7, 129.4, 136.3, 147.7, 163.1. IR (cm^-1^): ν 645, 723, 752, 784, 862, 909, 958, 1040, 1098, 1128, 1346, 1463, 1490, 1570, 1620, 2231, 2360, 2854, 2926, 3301. ESI-MS m/z: 380 ([M+H]**^+^**, 100). Anal. Calcd for C_26_H_37_NO, 1.6 H_2_0: C, 79.90, H, 9.86, N, 3.58. Found: C, 79.86, H, 9.40, N, 3.35. ESI-HRMS Calcd for [M+H]^+^ C_26_H_38_NO: 380.2953. Found: 380.2957.

**15-(quinolin-3-yl)pentadec-1-yn-3-ol (9e).** Yield: 54% (55 mg). ^1^H NMR (400 MHz): δ 1.25 (m, 20H), 1.70 (m, 2H), 1.82 (m, 2H), 2.44 (d, *J*= 2.1 Hz, 1H), 2.97 (t, *J*= 7.8 Hz, 2H), 4.37 (td, *J*= 2.1 Hz, *J*=6.6 Hz, 1H), 7.30 (d, *J*= 8.4 Hz, 1H), 7.47 (t, *J*= 7.8 Hz, 1H), 7.67 (td, *J*= 1.5 Hz, *J*= 7.2 Hz, 1H), 7.76 (d, *J*= 8.1 Hz, 1H), 8.06 (d, *J*= 8.4 Hz, 2H). ^13^C NMR (50 MHz): δ 25.0, 29.1, 29.4, 29.5, 30.1, 37.7, 39.2, 62.2, 72.6, 85.2, 121.4, 125.6, 126.7, 127.4, 128.6, 129.3, 136.3, 147.7, 163.1. IR (cm^-1^): ν 625, 749, 828, 1047, 1121, 1141, 1215, 1312, 1427, 1465, 1506, 1566, 1602, 1620, 1717, 2341, 2360, 2854, 2925, 3012, 3307. ESI-MS m/z: 352 ([M+H]**^+^**, 100). ESI-HRMS Calcd for [M+H]^+^ C_26_H_38_NO: 352.2640. Found: 352.2645.

**17-(quinolin-3-yl)heptadec-1-yn-3-ol (9f).** Yield: 62% (63 mg). ^1^H NMR (300 MHz): δ 1.28 (m, 22H), 1.69 (m, 2H), 1.80 (m, 2H), 2.45 (d, *J*= 2.1 Hz, 1H), 2.98 (t, *J*= 7.8 Hz, 2H), 4.37 (td, *J*= 2.1 Hz, *J*= 6.6 Hz, 1H), 7.30 (d, *J*= 8.4 Hz, 1H), 7.48 (t, *J*= 7.2 Hz, 1H), 7.69 (t, *J*= 8.1 Hz, 1H), 7.78 (d, *J*= 8.1 Hz, 1H), 8.07 (d, *J*= 8.1 Hz, 2H). ^13^C NMR (50 MHz): δ 25.0, 29.2, 29.5, 30.1, 37.7, 39.2, 62.2, 72.7, 85.2, 121.4, 125.6, 126.7, 127.4, 128.6, 129.3, 136.3, 147.7, 163.1. IR (cm^-1^): ν 561, 628, 668, 749, 828, 1049, 1215, 1427, 1464, 1506, 1565, 1602, 1620, 1707, 1968, 2159, 2854, 2926, 3019, 3306. ESI-MS m/z: 380 ([M+H]**^+^**, 100). ESI-HRMS Calcd for [M+H]^+^ C_26_H_38_NO: 380.2953. Found: 380.2957.

**icos-1-yn-3-ol (9g).** Yield: 50% (187.3 mg). ^1^H NMR (300 MHz): δ 0.88 (t, *J*= 7.5 Hz, 3H), 1.25 (s, 24H), 1.46 (m, 4H), 1.70 (m, 4H), 2.47 (t, *J*= 2.1 Hz, 1H), 4.37 (td, J= 1.8 Hz, *J*= 6.3 Hz, 1H). ^13^C NMR (50 MHz): δ 14.1, 22.7, 25.0, 29.2, 29.4, 29.5, 29.6, 29.7, 31.9, 37.7, 62.4, 72.8, 85.0. IR (cm^-1^): ν 735, 909, 1074, 1465, 2352, 2839, 2916, 3282. High resolution mass spectrometry led to decomposition EI-MS-m/z: 247(0.5), 233(0.5), 219(0.5), 205(0.5), 191(0.5), 177(0.5), 163(1), 149 (2), 135(5), 121(8), 107(8), 94(18), 81(22), 67(25), 54(40).

**S2 : Mesencephalic cultures**. Animals were treated in accordance with the Declaration of Helsinki and the Guide for the Care and Use of Laboratory Animals (U.S. National Institutes of Health). The embryos were removed at embryonic day 15.5 from pregnant Sprague-Dawley rats (Elevage Janvier, Le Genest St. Isle, France) that had been anesthetized, then decerebrated. The ventral mesencephalon was dissected as described below. Cell suspensions prepared by mechanical trituration without proteolytic enzymes were plated in 24 or 48 multiwell culture plates (Costar, Corning Inc., NY) precoated overnight with 1 mg/ml polyethylenimine (Sigma, Saint Quentin Fallavier, France) in borate buffer, pH 8.3. The cells were then maintained for maturation and differentiation in Neurobasal culture medium (Gibco, Invitrogen, Scotland, UK) supplemented with 1%_v:v_ B27 (Gibco, Invitrogen, Scotland, UK), 4 mM glutamine (Glutamax, Gibco, Invitrogen, Scotland, UK), 100 U/ml penicillin (Gibco, Invitrogen, Scotland, UK), and 100 μg/ml streptomycin (Gibco, Invitrogen, Scotland, UK). Cultures were treated at DIV 1 and every four days 300 µL of culture medium were replaced by medium supplemented with treatments.

**S3 : Immunofluorescence staining.** Tyrosine hydroxylase (TH) immunofluorescence was used to detect DA neurons. After 12 min fixation with a 4% formaldehyde solution in Dulbecco’s phosphate-buffered saline (PBS), cells were washed three times with PBS and then incubated in PBS^+^ (PBS containing 0.2% Triton X-100, 10% fetal bovine serum (Sigma, Saint Louis, MO) and 0.01% thimerozal (Sigma, Saint Quentin Fallavier, France) for 1 hour. The cells were further incubated overnight at 4°C with a rabbit anti-TH polyclonal antibody (1:500, Pel Freez, Paris, France) or a mouse anti-MAP-2 monoclonal antibody (1:100, AP-20, Sigma-Aldrich, Saint Quentin Fallavier, France). Subsequent incubations were performed, at room temperature, with a secondary anti-rabbit IgG cyanin 3 conjugate (1:500, Jackson immunoresearch Laboratories, Newmarket, UK) or an Alexa Fluor 488 F(ab’)2 fragment of goat anti-rabbit IgG (1:500, Invitrogen) or an anti-mouse IGg cyanin 3 conjugate (1:500, Jackson Immunoresearch Laboratories, Newmarket, UK). Concerning phospho-ErK1/2 (pp42/pp44) immunofluorescence staining, the cultures were incubated overnight at 4°C with a mouse monoclonal anti phospho- Erk1/2 antibody (Cell Signaling Technology Inc., Beverly, MA), diluted at 1:100 in PBS^+^ then washed and incubated with an anti-mouse IGg cyanin 3 conjugate (1:500, Jackson Immunoresearch Laboratories, Newmarket, UK). Images were acquired with Simple-PCI software from C-Imaging Systems using an inverted fluorescent microscope (TE-300, Nikon, Tokyo, Japan) coupled to an ORCA-ER digital camera from Hamamatsu (Bridgewater, NJ).

**S4 : Survival quantification.** Tyrosine hydroxylase (TH) immuno cytochemistry was used to quantify the survival of DA neurons. Ten randomly chosen fields per well were counted. Results were expressed in percentage of untreated cultures. Each value represents the mean ± S.E.M. from three wells per condition of three independent experiments.

**S5 : Neuritogenesis quantification**. Neurite length per cell was quantified using the image analysis software Neurite Outgrowth (Explora Nova, La Rochelle, France) on at least 100 neurons randomly chosen per condition. Results were expressed as percentage of untreated cultures. Each value represents the mean ± S.E.M. from three wells per condition of three independent experiments.

**S6 : MPP^+^ intoxication**. Dissociated ventral mesencephalons were cultured as previously described in presence or absence of compound **6c**. At DIV5, 300 µL of 500 µL medium were replaced by fresh medium supplemented or not with compound **6c** at 10, 100, 1000 nM and 2, 3 or 4 µM of MPP^+^ (Sigma, Saint Quentin Fallavier, France) were added. At DIV 7, medium containing MPP^+^ was removed completely then replaced by fresh medium supplemented or not with compound **6c**. At DIV 8, cells were fixed for 12 min with a 4% formaldehyde solution, washed three times with 500 µL PBS then submitted to TH immunostaining to allow analysis of DA neurons. Each value represents the mean ± S.E.M. from three wells per conditions of three independent experiments.

**S7 : Uptake of neurotransmitters**. High-affinity uptake of [^3^H]-DA and [^3^H]-GABA (GE Healthcare, Little Chalfont, Buckinghamshire, UK) was determined according to a modified method described by Alexi and Hefti (1). Cells were washed three times with the incubation solution (5 mM glucose in Dulbecco’s phosphate-buffered saline (D-PBS), pH 7.4), preincubated for 10 min at 37°C with 500 µL of incubation solution, before adding 50 nM [^3^H]-GABA (88 Ci/mmol) or [^3^H]-DA (40 Ci/mmol) for a further 5 min (GABA) or 15 min (DA) period. Blanks were obtained by incubating the cells at 4°C (GABA) or by using GBR-12909 (Sigma, Saint Quentin Fallavier, France) at 5 µM (DA). Uptake was stopped by removing the incubation mixture, followed by three rapid washes with ice-cold PBS. Then, 500 μl of distilled water were added and cells were scraped. The extracted radioactivity was measured by liquid scintillation spectrometry after addition of 7 ml scintillation cocktail (BCS, Amersham Biosciences, Little Chalfont, Buckinghamshire, UK) to each vial. Each value represents the mean ± S.E.M. from three wells per condition of three independent experiments.

**S8 : Uptake of [methyl-^3^H]thymidine.** A marker of DNA synthesis, [methyl*-*^3^H]-thymidine (GE Healthcare, Little Chalfont, Buckinghamshire, UK), was used to label proliferating cells as described previously (2). Mesencephalic treated cultures with 10, 100 or 1000 nM of compound **6c**, or 2 µM arabinoside-C (ARA-C) or 20 ng/mL epidermal growth factor (EGF) were exposed to 1 µCi of [methyl-^3^H]-thymidine (40 Ci/mmol) at 37°C for 18 hours. After three washes with culture medium, the cells were fixed with 4% formaldehyde for 12 min. Positive nuclei were visualized with Hypercoat LM-1 emulsion (GE Healthcare, Little Chalfont, Buckinghamshire, UK) after 10 days exposure at 4°C then quantified by counting 15 fields photographed per well. Each value represents the mean ± S.E.M. from three wells per condition of three independent experiments expressed as a percentage of untreated cultures values.

**S9 : Western Immunoblotting of Akt and ERK1/2.** Cells were lysed in lysis buffer (Tris HCl pH 7.4 50 mM, NaCl 150 mM, EDTA 4 mM, Triton X100 0.2%, nonyl phenoxylpolyethoxylethanol (NP40) 0.3%, in water) and clarified (13000 rpm, 15 min, 4°C), and protein concentration was determined with the Bradford assay (Bio-Rad). Boiled lysates supplemented with Laemmli buffer containing 1 mM sodium orthovanadate, 1 µM okadaic acid, and 0.5% β-mercaptoethanol were separated in precast 4-12% Tris-glycine SDS-polyacrylamide gels (Gibco, Invitrogen, Scotland, UK), and proteins were transferred to nitrocellulose membrane. After blocking with 10% BSA then 10% milk for 30 min each at room temperature, membranes were incubated overnight at 4°C with a rabbit polyclonal anti phospho- Erk1/2 antibody (1:1000; Cell Signaling Technology Inc., Beverly, MA) and 2 hours at room temperature with anti-rabbit horseradish peroxidase-conjugated secondary antibody at 1:1000 (Pierce Chemical, Rockford, IL). The immune complexes were visualized by enhanced chemiluminescence (Pierce Chemical, Rockford, IL). The immunostaining of α-actin was performed using a rabbit anti-α-actin antibody (1:1000, Sigma, Saint Quentin Fallavier, France) on the same membrane after stripping using the Re-Blot Plus kit from Chemicon International (Temecula, CA). All immunoblots were repeated for at least three independent experiments and density of bands was quantified using Image J software.

**S10: Statistical Analysis.** Comparisons between two groups were performed with Student’s *t* test. Multiple comparisons against a single reference group were made by one-way analysis of variance (ANOVA) followed by Dunnett’s or Bonferroni’s test. When all pairwise comparisons were made, two-way ANOVA was used followed by Duncan’s test. S.E.M. values were derived from at least three values per condition of three independent experiments.

## References

**(1)** Alexi T & Hefti F (1993) Trophic actions of transforming growth factor alpha on mesencephalic dopaminergic neurons developing in culture. *Neurosc* 55(4):903-918

**(2)** Troadec JD, Marien M, Mourievat S, Debeir T, Ruberg M, Colpaert F & Michell PP (2002) Activation of the mitogen-activated protein kinase (ERK(1/2)) signaling pathway by cyclic AMP potentiates the neuroprotective effect of the neurotransmitter noradrenaline on dopaminergic neurons. *Mol. Pharmacol* 62(5):1043-1052
